# Prediction of Redox Power for Photocatalysts: Synergistic Combination of DFT and Machine Learning

**DOI:** 10.1021/acs.jctc.3c00286

**Published:** 2023-06-29

**Authors:** Péter Pál Fehér, Ádám Madarász, András Stirling

**Affiliations:** † Institute of Organic Chemistry, Research Centre for Natural Sciences, Magyar tudósok körútja 2, 1117 Budapest, Hungary; ‡ Department of Chemistry, Eszterházy Károly University, Leányka u. 6, 3300 Eger, Hungary

## Abstract

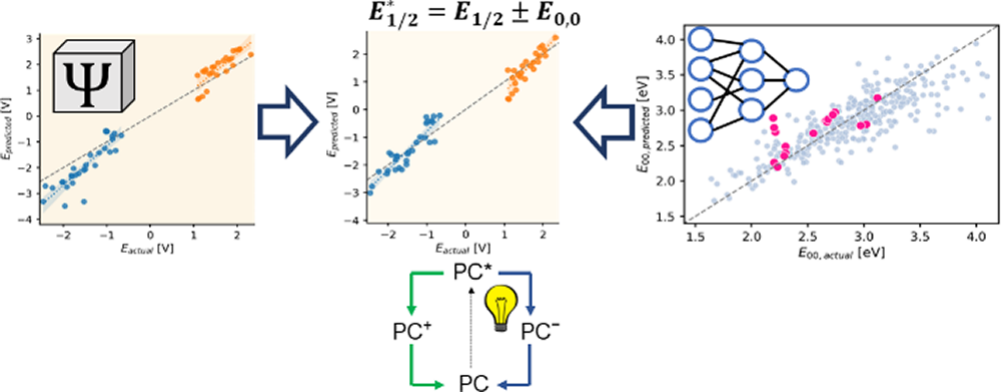

The accurate prediction of excited state properties is a key element of rational photocatalyst design. This involves the prediction of ground and excited state redox potentials, for which an accurate description of electronic structures is needed. Even with highly sophisticated computational approaches, however, a number of difficulties arise from the complexity of excited state redox potentials, as they require the calculation of the corresponding ground state redox potentials and the estimation of the 0–0 transition energies (*E*_0,0_). In this study, we have systematically evaluated the performance of DFT methods for these quantities on a set of 37 organic photocatalysts representing 9 different chromophore scaffolds. We have found that the ground state redox potentials can be predicted with reasonable accuracy that can be further improved by rationally minimizing the systematic underestimations. The challenging part is to obtain *E*_0,0_, as calculating it directly is highly demanding and its accuracy depends strongly on the DFT functional employed. We have found that approximating *E*_0,0_ with appropriately scaled vertical absorption energies offers the best compromise between accuracy and computational effort. An even more accurate and cost-effective approach, however, is to predict *E*_0,0_ with machine learning and avoid the use of DFT for excited state calculations. Indeed, the best excited state redox potential predictions are achieved with the combination of M062X for ground state redox potentials and machine learning (ML) for *E*_0,0_. With this protocol, the excited state redox potential windows of the photocatalyst frameworks could be adequately predicted. This shows the potential of combining DFT with ML in the computational design of photocatalysts with preferred photochemical properties.

## Introduction

Photoredox catalysis has gained significant attention in chemical synthesis over the last decade due to its effectiveness in the activation of small molecules.^1^ The underlying mechanism is mostly single-electron transfer (SET),^2^ for which the photocatalysts (PCs) provide easy access.^3^ This type of electron transfer reactivity is described by the reduction potentials of redox couples, which can be written as follows:
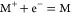
1
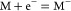
2

**Scheme 1:**
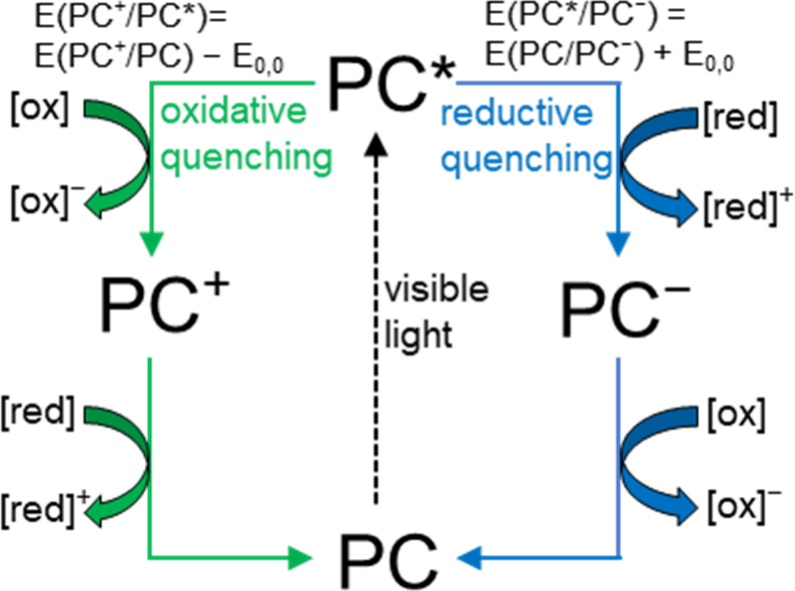
Typical Photoredox Cycles

We use the notation *E*(M^+^/M) and *E*(M/M^–^) for the redox potentials of eqs 1 and 2, respectively, and omit the designation of any radical species in general expressions. To have a basic understanding about their values and relation, we can assume that eq 1 is thermodynamically more favored, so the value of *E*(M^+^/M) is more positive than *E*(M/M^–^) due to the negative correlation (eq 3) with the reaction free energy Δ*G*^0^.
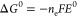
3Here, *n*_e_ is the number of electrons participating in the redox process, *F* is the Faraday constant, and *E*^0^ is the electromotive force. The key feature of photoredox catalysis is that we can exploit the increased reactivity of the excited state. As the absorption of visible light provides more energy than the energy required to reduce or oxidize the ground state, replacing **M** in eqs 1 and 2 with its excited state (M*) inverts the energetics of the reactions. These reactions are then able to drive otherwise unfavorable SET processes. Taking away the energy of the excited state in this way is called quenching, and it may involve oxidation or reduction, as shown in Scheme 1. In the oxidative quenching mechanism, the excited photocatalyst (PC*) is oxidized in a step called photoinduced electron transfer (PET), and then the oxidized photocatalyst (**PC^+^**) is reduced to the initial PC in the turnover step. Note that both SET half reactions involving the catalyst are expected to be thermodynamically favored; therefore, the energy of the light is distributed into two otherwise not spontaneous reactions. Usually, one of these involves the substrate, while the other is either the reduction of molecular oxygen or the oxidation of a tertiary amine (e.g., TEA) additive. The value of the *E*(O_2_/O_2_^·–^) potential is −0.87 V vs saturated calomel electrode (SCE) (in MeCN),^4^ while the *E*(TEA^·+^/TEA) potential is +0.78 V vs SCE (in MeCN).^5^ The reductive quenching cycle is conceptually the same with inverted redox behavior in the PET and turnover steps.

**Scheme 2:**
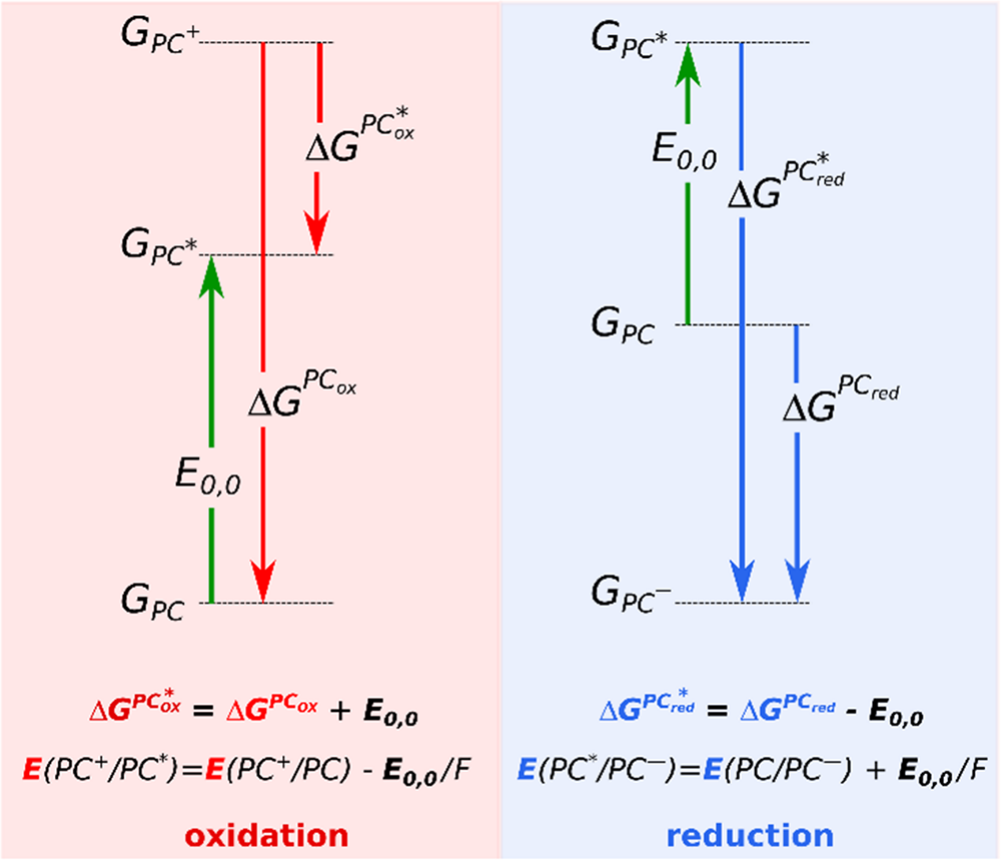
Diagrammatic Representation of Ground State and Excited State Redox Gibbs Free Energies and the Corresponding Redox Potentials The direction of the arrows corresponds to the definition of the given energy term. Note that the redox potentials are consistently defined as reduction half reactions irrespective of whether they actually correspond to electron donation or acceptance. *E*(ox/red)-s are the redox potentials, and they correspond to *G*-s via eq 1; now *n*_e_ = 1; *G*-s are the Gibbs free energies; *E*_0,0_ is the 0–0 transition energy. The absolute redox potentials indicate that in each redox pair, the reduced form is always lower in Gibbs free energy than the oxidized form.^6^

**Scheme 3:**
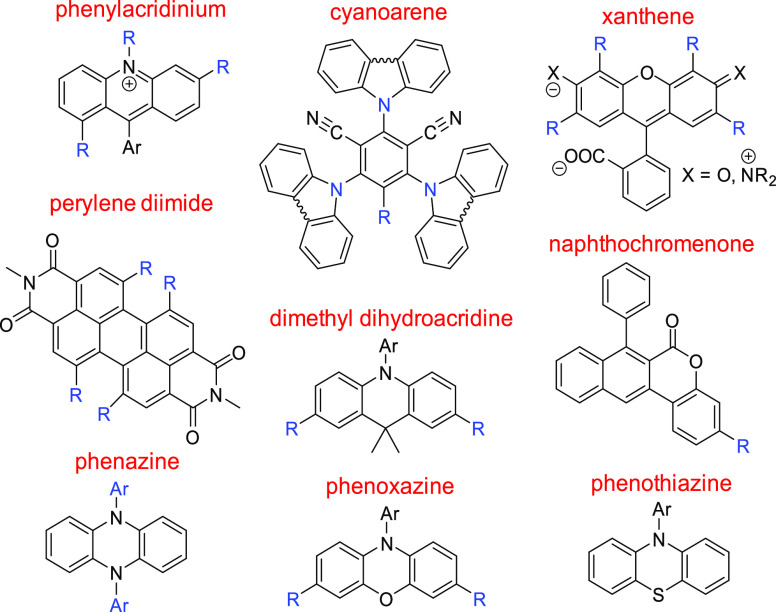
Nine Molecular Frames in the Photocatalyst Dataset The structures of the individual molecules can be found in ref (10).

In this work, we are interested in the excited state redox potentials of PCs. These can be calculated from the ground state redox potential and the 0–0 transition energy (*E*_0,0_), as shown in Scheme 2.^7^ In practice, *E*_0,0_ is usually determined from the intersection of the normalized absorption and emission spectra,^1b^ but it can be obtained directly from vibrationally resolved electronic spectra.^8^ We can also approximate *E*_0,0_ with the peak maximum of the lowest energy transition in the absorption spectrum, i.e., with the vertical absorption energy of the S_1_ state. This latter approach is evidently erroneous, but it allows for a rapid screening of molecules using time-dependent density functional theory (TDDFT). In calculations, however, the accuracy of the *E*_0,0_ estimation is influenced by additional factors like the DFT functional or solvation approximations.^9^ Our goal is to assess the impact of these factors and use this knowledge to propose an efficient computational protocol to calculate the working redox range of PCs, which can be used to determine the optimal catalysts for a given reaction. To this end, we first evaluate the performance of 15 DFT functionals at reproducing experimental ground state redox potentials for the set of organic PCs we compiled in a previous paper.^10^ The molecular frames of these OPCs are shown in Scheme 3.

Next, we use these potentials and the absorption database from ref (10) to calculate excited state redox potentials and discuss the possibilities for improving the accuracy. The best performing methods are then used to determine the redox window available for each PC. In practice, measuring these redox windows can be challenging due to the complexity of cyclic voltammograms that exhibit irreversibility, coupled chemical reactions, and multielectron processes.^11^ These difficulties lead to the fact that the reference database we compiled has missing data [i.e., either *E*(M^+^/M*) or *E*(M*/M^–^)] for some molecules. Furthermore, we can safely assume that not being able to measure, e.g., the *E*(M^+^/M*) potential does not necessarily mean that the PC cannot be used in the corresponding redox reaction. This is one of the areas where computations can offer an advantage, as we can calculate the data to fill in the holes in any redox database.

## Computational Methods

The ground state redox potentials were calculated from the reaction free energies of the redox half reactions (eqs 1 and 2) using the Nernst equation (eq 3). The free energies of both species in each redox couple were determined using eq 4:
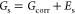
4where the s index refers to the solution phase and *G*_corr_ is the thermal correction to the Gibbs free energy determined in solution phase using the ideal gas-rigid rotor-harmonic oscillator approximation in standard conditions (*T* = 298.15 K and *c* = 1 mol/dm^3^). *E*_s_ is the electronic energy in the solution phase using the def2-TZVPP basis set.^12^ Geometries were optimized with the def2-SVP basis set applying the solvation model based on density.^12,13^ The use of additional diffuse functions was also evaluated, and it was found that their effect is insignificant (0.05 V or less). In the calculation of reaction free energies for the half reactions, the free energy of the electron was set to zero. To obtain the calculated redox potential for a half reaction in solvent S against the SCE (*E*_ox/red, S_^SCE^), the following form of the Nernst equation can be used:
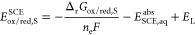
5where *E*_SCE, aq_^abs^ is the absolute potential of the aqueous SCE (+4.522 V), Δ_r_*G*_ox/red,S_ is the corresponding Gibbs free energy change, and *E*_L_ is the intersolvent potential (0.093 V for MeCN, 0.172 V for DMF).^6^ As *E*_L_ is not provided for dichloromethane (DCM) in the literature, a value of 0.0 V was used. Note that this choice is based on the observation that *E*_L_ correlates with the dipole difference relative to water and DCM has a dipole moment very close to that of water.^14^

The methodology mentioned above was used to benchmark the following set of functionals: PBE,^15^ B97D3,^16^ TPSS,^17^ M06L,^18^ M06,^19^ M062X,^19^ B3LYP,^20^ CAM-B3LYP,^21^ ωB97XD,^22^ B2PLYP,^23^ ωB2PLYP,^24^ DSD-BLYP,^25^ B2GP-PLYP,^26^ ωPBEPP86,^27^ and (SCS-)PBE-QIDH.^27,28^ Empirical dispersion corrections were added when these corrections are not built into the functionals. In these cases, we have employed the D3 correction of Grimme with Becke-Johnson damping.^16,29^ The calculations involving the double hybrid functionals [B2PLYP, ωB2PLYP, DSD-BLYP, B2GP-PLYP, ωPBEPP86, and (SCS-)PBE-QIDH] were carried out using the ORCA 5.0.2 software package.^30^ The parentheses in (SCS-)PBE-QIDH indicate that the spin-component scaling is applied for the excited states only, whereas for ground states, the original PBE-QIDH is employed. These six functionals were only used to obtain electronic energies and all calculations utilized the resolution-of-identity (RI) approximation.^31^ The geometries and the *G*_corr_ values were taken from M06L calculations. For the pure and hybrid functionals, geometry optimizations and frequency calculations were performed using the Gaussian16 suite.^32^

The DeepChem^33^ deep learning library of Python3 together with the freely available Deep4Chem^34^ database was used to design a machine learning (ML) approach that predicts *E*_0,0_ values. Three datasets were generated from the database: one for each solvent in our PC data. This way, three datasets were obtained with approximately 1700, 2300, and 600 entries for acetonitrile, DCM, and *N*,*N*-dimethylformamide, respectively. The chromophores in the database are given as SMILES strings, which were converted into 2048-bit numerical representations via the Morgan fingerprint transformer of DeepChem. The *E*_0,0_ values were calculated by averaging the tabulated absorption and emission maxima, and then, a scaling into the 0 to 1 range was applied. The data were split randomly into train and test sets at an 80:20 ratio. The fitted model is defined as a sequential neural network with a single hidden layer of 100 neurons employing ReLU activation together with a 50% dropout to prevent overfitting during training. We have used the Adam optimizer with a learning rate of 0.001 and set the number of epochs to 20 for training. We used grid search to determine the optimal number of neurons in the hidden layer, learning rate, and number of epochs that yield the lowest mean absolute error (MAE). After training, the model was able to predict *E*_0,0_ with MAE around 0.2 V for both the test set and our organic PC molecules. The code and additional details can be found on GitHub.^35^

## Results and Discussion

### Our Strategy in General

Scheme 2 indicates that the determination of excited state redox potentials requires the calculation of ground state redox potentials and the 0–0 transition energy. The ground state redox potentials are obtained via the calculation of Gibbs free energies of the oxidized and reduced forms of each PC, as indicated by eq 5. This process is straightforward and has been explored by others for different sets of molecules.^36^ It has been concluded that highly sophisticated quantum chemistry methods offer no advantages as the accuracy of the potentials is limited to around 0.3 V (MAE) when implicit solvation approximation is employed.^37^ We expect this performance from the best performing functionals here as well, so we only provide a brief analysis of functional performance and will focus more on the solvation effects present in our molecule set.

The *E*_0,0_ component is more challenging to obtain as it involves excited state (TDDFT) calculations, which are not only less accurate than ground state ones, but the differences between DFT functionals are also amplified.^10,38^ In addition, the direct calculation of *E*_0,0_ is highly demanding, so in practice, additional approximations are used. For example, using vertical absorption energies (*E*_abs_) in place of *E*_0,0_ has an error around 0.22–0.27 eV based on experimental data (Figure S1). However, it is a systematic overestimation, which can be reduced via empirical shift or scaling, as shown in Figure S2. Therefore, we first present excited state redox potential predictions with the *E*_abs_ estimate for *E*_0,0_, and then, we discuss the possibility of improving this approach. The intricacies of the theoretical prediction of *E*_0,0_ imply that presently this is not an ideal approach for the practice. We therefore also set out to explore ML-based predictions. This approach involves the training of neural network-based models on thousands of datapoints offered by the recently published Deep4Chem database.^34^

### Ground State Redox Potentials

**Figure 1:**
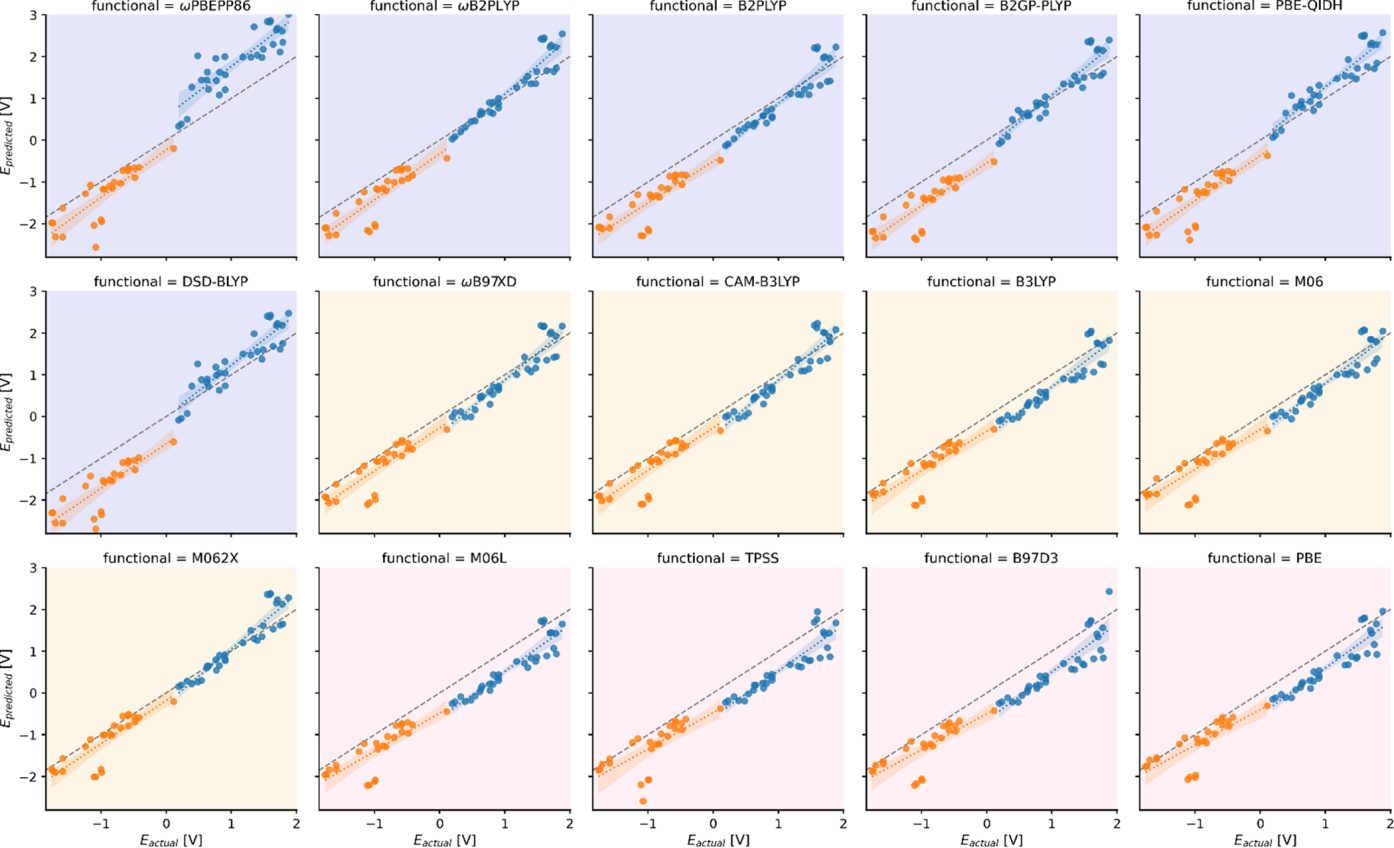
Calculated vs reference ground state redox potentials (vs SCE). The dashed lines in gray indicate the perfect prediction. The blue and orange colors correspond to the *E*(M^+^/M) (oxidation) and *E*(M/M^–^) (reduction) potentials, respectively. The colored backgrounds are used to differentiate double hybrid (violet), hybrid (yellow), and pure DFT functionals (light rose).

The results of the 15-functional benchmark for the calculation of the *E*(M^+^/M) and *E*(M/M^–^) potentials are shown in Figure 1. It is apparent that the reference values are systematically underestimated with the only exceptions being the *E*(M^+^/M) potentials predicted by some double hybrids. Another noticeable feature is a group of outliers near *E*_actual_ = −1.0 V, for which all functionals yield errors above 1 V. These points correspond to the reduction of eosins, where their charge drops from −2 to −3, the largest absolute charge value in our dataset. A similar effect can be observed in the positive potential region, where the six BOH-Acr and BF3-Acr molecules are oxidized from +1 to +2 charge. Note that these acridines are outliers with respect to the rest of the calculated points, and for most functionals, they have low errors due to error cancellation.

**Figure 2:**
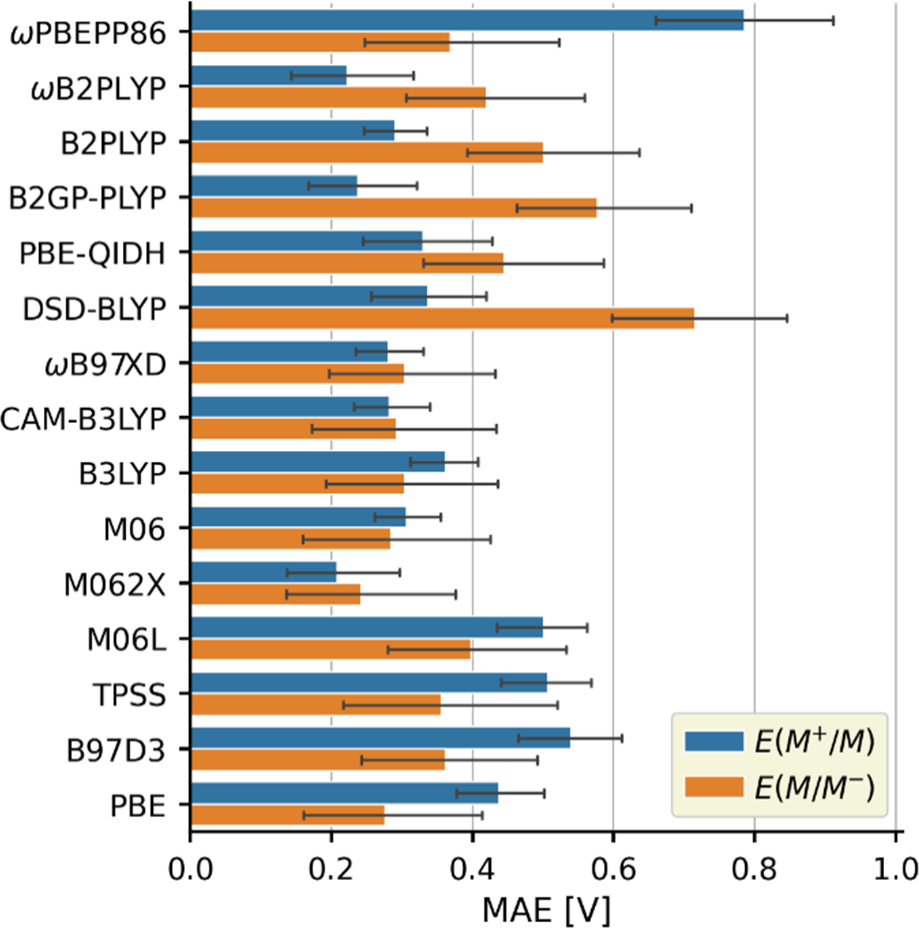
MAEs of the ground state redox potential predictions. The error bars correspond to 95% confidence intervals.

For further analysis, we collected the MAEs of the predictions in Figure 2. The overall MAE is 0.38 V. In general, the hybrid functionals are more accurate than the pure functionals, while the double hybrids yield mixed result with good *E*(M^+^/M) and poor *E*(M/M^–^) performance, except for ωPBEPP86. The predictions given by double hybrids also exhibit contrasting variances as the points in Figure 1 scatter noticeably more for ωPBEPP86, PBE-QIDH, and DSD-BLYP compared to the functionals based on the B2PLYP scheme. Overall, the best performing functional is M062X with an average MAE of 0.21 and 0.24 V for *E*(M^+^/M) and *E*(M/M^–^), respectively. The range-separated hybrids CAM-B3LYP and ωB97XD also offer a balanced and reasonably good accuracy with an MAE of around 0.29 V. The double hybrids offer no significant advantage over hybrid functionals, especially for *E*(M/M^–^), so the added computational effort to use them is not justified. Among the computationally least demanding pure functionals, PBE performs the best with MAEs of 0.44 and 0.28 V for *E*(M^+^/M) and *E*(M/M^–^), respectively. The good *E*(M/M^–^) accuracy makes PBE ideal for screening studies, but its use for *E*(M^+^/M) should be avoided.

As we have seen, the best performing functionals indeed approach the accuracy limit set by the implicit solvent model.^37^ Therefore, to further improve the results, we need to understand better the solvation effects present in our benchmark set of PCs. We use the B2PLYP functional here, but note that the choice of the functional is expected to have a negligible effect on the results (Figure S3). First, we have compared the reaction free energies (Δ*G*_r_) with the reaction solvation energies (i.e., Δ*E*_solv_ = *E*_solv_[*M*] – *E*_solv_[M^+^] for eq 1, where *E*_solv_[X] is the solvation energy of species X) for the reduction and oxidation of all molecules. Note that Δ*E*_solv_ is a component of the reaction free energy.

**Figure 3:**
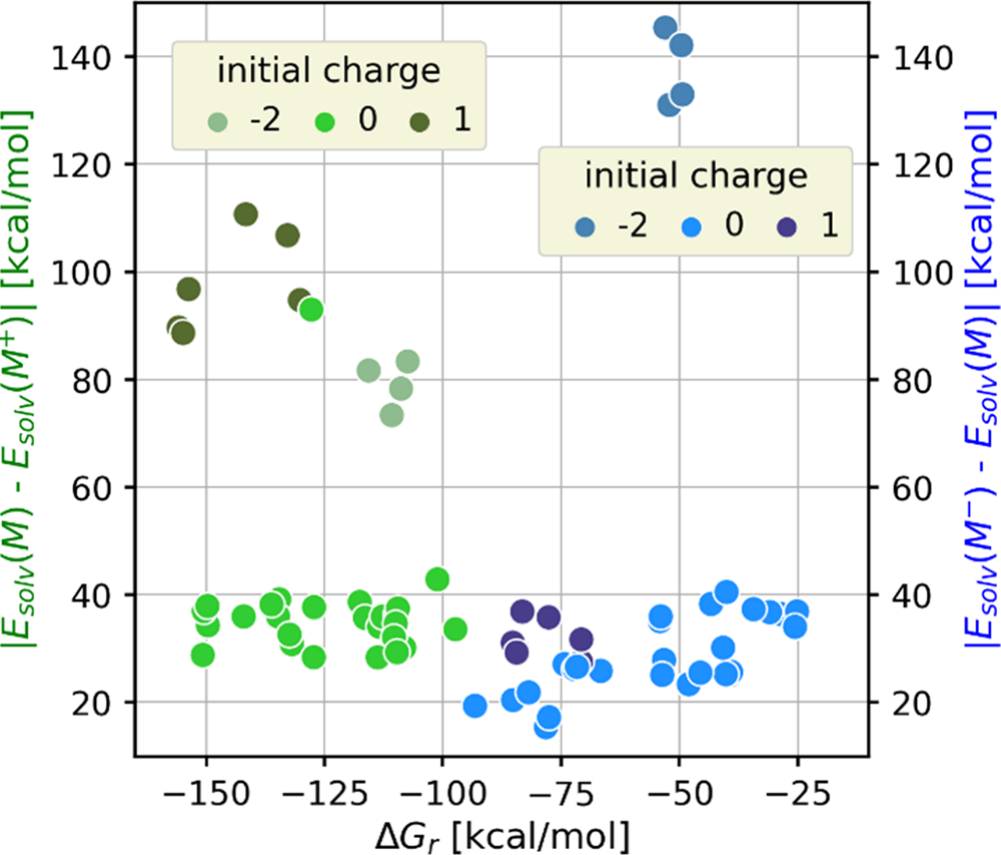
Distribution of |Δ*E*_solv_|−Δ*G*_r_ pairs calculated with the B2PLYP functional. The coloring indicates the charge of the initial form of the PC in the oxidation (green hue) and reduction (blue hue) half reactions.

The results shown in Figure 3 indicate that the differences in the Δ*E*_solv_ values primarily originate from the charges of the species involved in the reaction and that the molecular composition is less important. The absolute values of Δ*E*_solv_ are also an indication of the strength of the solvent effect: we can assume that the error of the implicit solvent model is more pronounced when |Δ*E*_solv_| is large. The solvent error is even more severe in the upper right region of Figure 3, where the |Δ*E*_solv_| values are large and Δ*G*_r_ small, i.e., Δ*E*_solv_ dominates the calculated potentials for these half reactions. Not surprisingly, this region is populated by the reductions of eosins that appear as outliers in Figure 1. One way to compensate this error is to introduce counterions such that the initial **M** form of the molecules has zero charge. Indeed, Tables S1 and S2 show that the redox potentials of the eosins recalculated with this approach show significant improvement. For example, the MAEs calculated by M062X for the *E*(M/M^–^) potential are reduced from around 1 to 0.75 V and 0.5 V when one and two sodium ions are added, respectively. However, there is still a considerable discrepancy between theory and experiment that necessitates additional correction.

**Figure 4:**
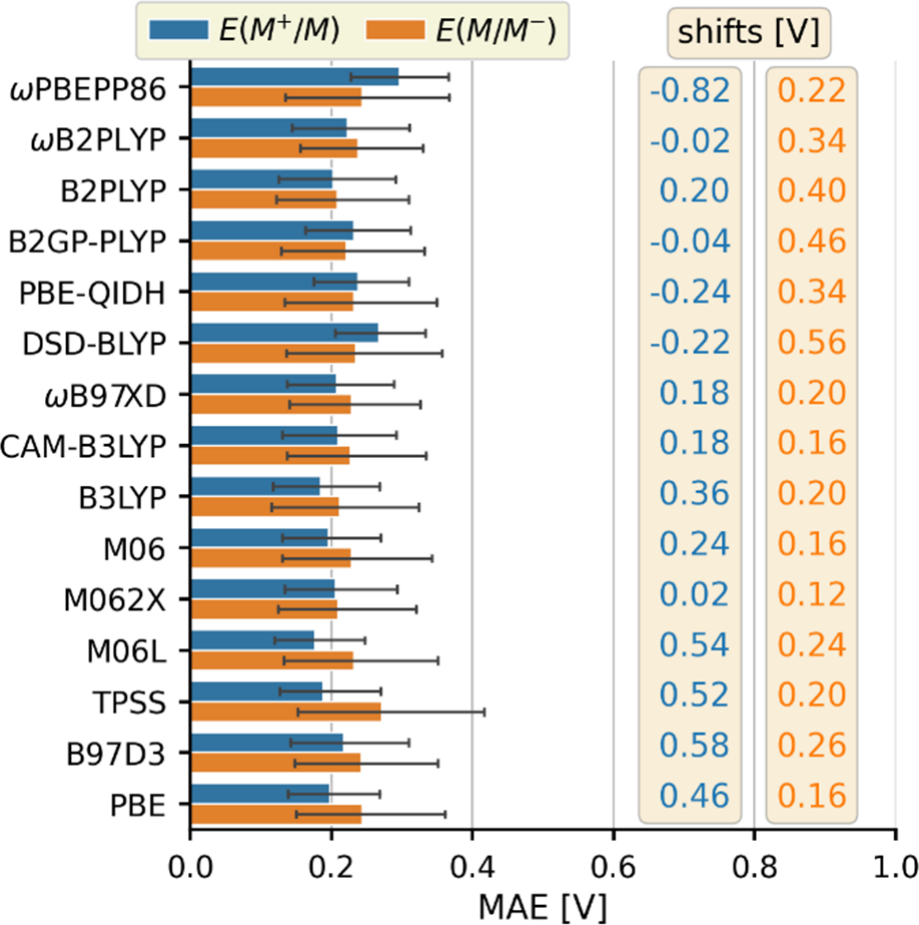
MAEs of the ground state redox potential predictions after using the optimized shift parameters on the right.

The effect of counterions can also be simulated by adding a charge-dependent correction term based on the generalized Born theory to the redox potential.^39^ This pseudo counterion method, however, involves parameter fitting, so its advantage over conventional data-driven (non-)linear corrections is mostly its physics-based explainability. Therefore, we first evaluate the improvements offered by a shift of the calculated redox potentials, as we are interested in finding the simplest possible approach that provides reasonable accuracy. Based on Figures 1 and 3, this model compensates the error from the change of one unit of charge in oxidation or reduction, which is the main source of the systematic bias exhibited by all functionals. Note that the shift correction is equivalent to adjusting the potential of the reference electrode, which is a common interpretation of this approach.^37e,39^ To this end, we have determined optimal shift values that yield the lowest possible MAE individually for all functionals. Figure 4 shows that this adjustment removes most of the differences between the functionals and yields an overall MAE of 0.22 ± 0.01 V. The analysis of the shift values in Figure 4 reveals that M062X offers the best performance, as it requires the smallest adjustments, while CAM-B3LYP and ωB97XD are the most consistent, as optimization yields relatively small shift values that differ by only 0.02 V between the two potentials. In contrast, the worst performing double hybrids (ωPBEPP86 and DSD-BLYP) and all pure functionals require a shift of at least 0.56 V for one of the potentials. For our search of a general potential prediction approach, however, the use of functional-specific parameters is undesirable. Therefore, we have also determined an optimal universal shift of 0.2 V that yields a slightly worse overall MAE of 0.32 ± 0.01 V together with an average MAE of 0.23 ± 0.01 V for the hybrid functionals (Figure S6 and Table S3).

The errors can be further reduced via linear scaling, as shown in Figures S4 and S5, or by introducing additional charge dependent fitting parameters to handle the outliers. However, we provide no further discussion about this topic, as not only is it outside the scope of the present work but also because there are several recent reports where the authors go even beyond linear corrections: (a) reduction potentials for about 315k carbonyl-to-alcohol and carbonyl-to-amine reactions of seed metabolites were calculated using the PM7 semiempirical method and then corrected with Gaussian process regression.^40^ The model was evaluated on 81 experimental redox potentials and yields an MAE of 23 mV in the −350 to −100 mV range, indicating a similar relative error compared to our results. (b) A slightly higher MAE of 36 mV in the −400 to 100 mV range was obtained for the prediction of midpoint redox potentials of 141 flavoproteins using a fully ML (extreme gradient boosting, XGB) approach trained on structural descriptors.^41^ (c) B3LYP-D3-calculated redox potentials in implicit solvent were improved from MAE = 0.43 to 0.22 V in the −3 to 2 V redox range for the 193 organic molecules of the ROP313 database using kernel ridge regression.^37a^ This is essentially the same error that we have obtained in Figure 4. The authors also performed calculations using a microsolvated cluster approach but found no improvement over the implicit solvent model in terms of MAE. Example (b) also demonstrates the possibility of using ML alone^41b^ to predict redox potentials; however, the training set of 141 flavoproteins seems to be too small and too specific for a more general model. Being too specific is an issue even for large computational databases like RedDB, which contains about 32k molecules limited to certain quinone and aza-aromatic cores.^42^ Therefore, we do not attempt to include pure ML models for redox potential prediction in the present study.

### Excited State Redox Potentials

**Figure 5:**
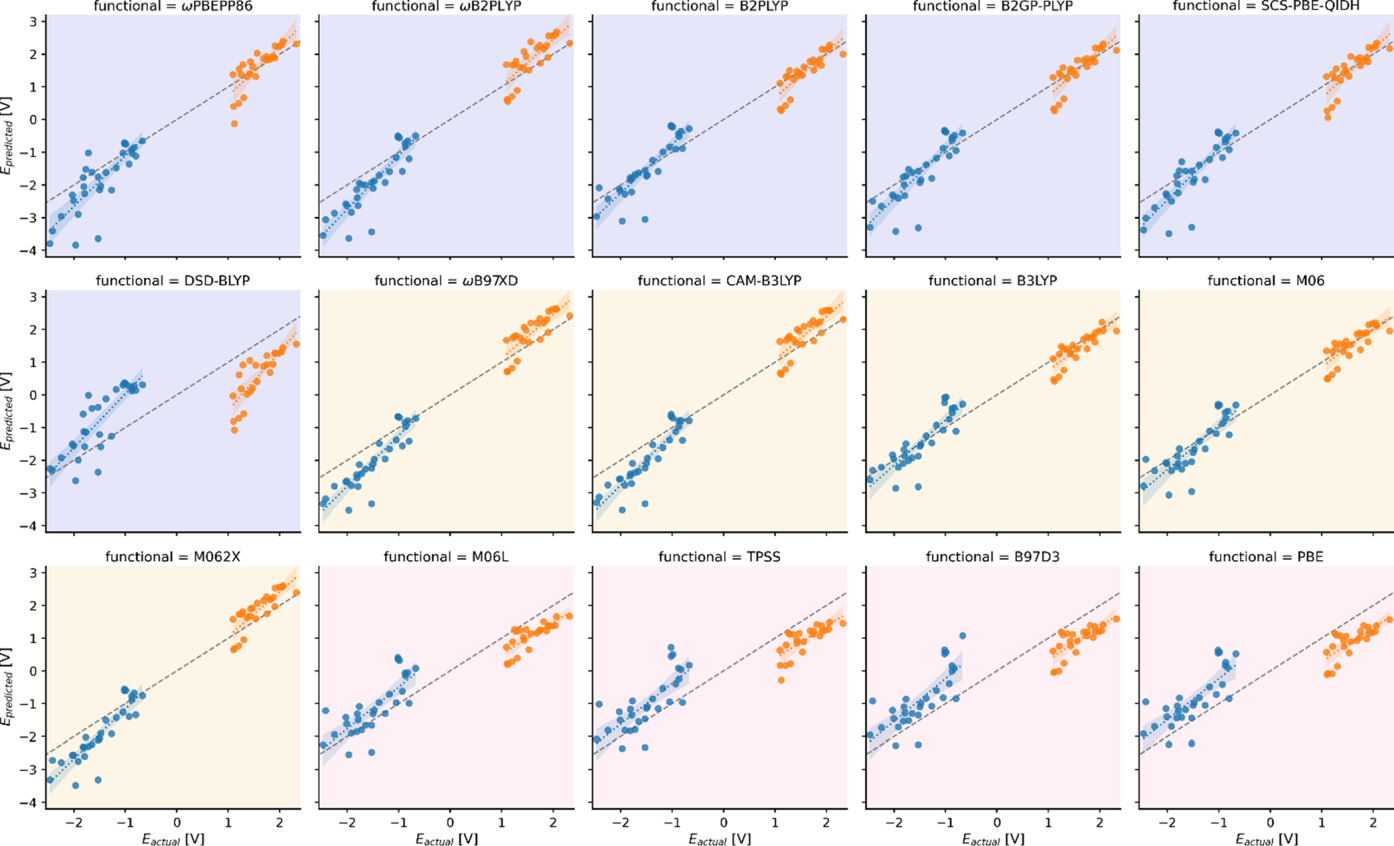
Calculated vs reference excited state redox potentials. The dashed lines in gray indicate the perfect prediction. The blue and orange colors correspond to the *E*(M^+^/M*) and *E*(M*/M^–^) potentials, respectively.

**Figure 6:**
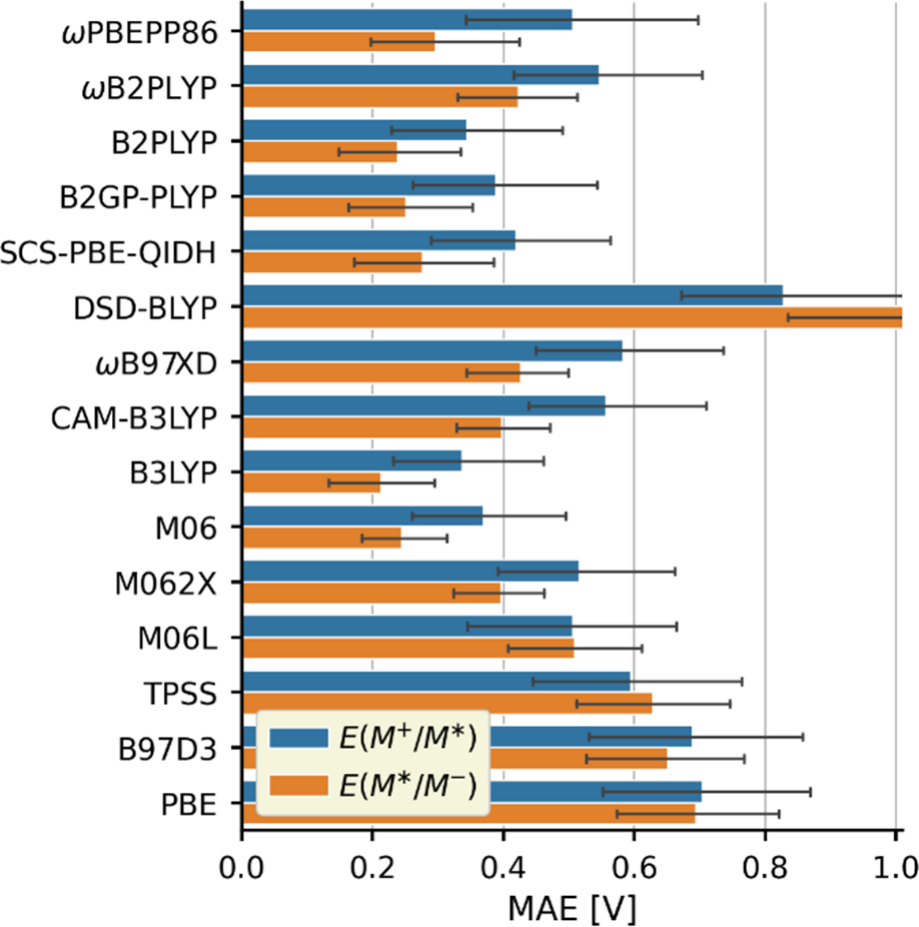
MAEs of the calculated excited state redox potentials. The error bars correspond to 95% confidence intervals.

The excited state redox potentials are calculated using the two formulae in Scheme 2. A key issue here is to obtain the *E*_0,0_ values. The simplest approximation is to use the first vertical absorption energy (*E*_abs_). We have found that when the original DFT ground state redox potentials and the *E*_abs_ values are combined, the results are inconsistent (Figures S7 and S8). We can improve this approximation by offsetting the overestimation introduced by using *E*_abs_ instead of *E*_0,0_. Figure S2 indicates that the initial MAE of 0.27 eV can be reduced to 0.11 eV when a scaling factor of 0.91 is applied to the *E*_abs_ values. Note that we also evaluated the use of a shift parameter (−0.26 eV), but it yielded inferior MAE (0.14 eV). Therefore, we have employed the scaled *E*_abs_ (*E*_0,0_ = 0.91 × *E*_abs_) approximation to obtain Figures 5 and 6, which show the results of the 15-functional benchmark for the calculation of the *E*(M^+^/M*) and *E*(M*/M^–^) potentials. The ground state component of the potentials was also improved by shift values introduced in Figure 4 (Figures S9 and S10 show the results without this correction). Note that the *E*_abs_ values were taken from ref (10), except for the acridines which were recalculated using DCM solvent to match experiment.

The reference data on the horizontal axes in Figure 5 show that the two types of potentials are separated by an approximately 1.5 V gap. This is to be expected, as most molecules absorb around 2.5 eV (ca. 500 nm), which transforms the highest *E*(M^+^/M) around 2.0 V to *E*(M^+^/M*) = −0.5 V, and the lowest *E*(M/M^–^) values near −1.5 V to *E*(M*/M^–^) = +1.0 V. Also, the molecules under investigation here are proven PCs that are potent oxidant or reductants in the excited state, so they are designed to have at least one potential with a large absolute value. The horizontal potential gaps, however, are reproduced by the predicted gaps (read vertically) rather poorly in most cases. In fact, they are reproduced reasonably well by only the three hybrid functionals (M062X, CAM-B3LYP, and ωB97XD) that also offered good ground state performance. The lack of the gap in the other cases is due to the inconsistent description of the two types of potentials. This issue is most pronounced for DSD-BLYP and for the pure functionals. Regardless of their ability to reproduce the gap, the MAEs in Figure 6 indicate that the functionals that perform well in TDDFT calculations (B2PLYP, B2GP-PLYP, SCS-PBE-QIDH, and M06) for this molecule set offer better excited state redox potentials than those functionals with good ground state redox potentials but poorer TDDFT performance (DSD-BLYP, ωB97XD, CAM-B3LYP, and pure functionals).^10^ This is not surprising since the applied functional-dependent ground state potential shifts remove most of the differences originating from the ground state. Note that a universal 0.2 V shift leads to mostly the same conclusions (Figures S11 and S12). Interestingly, the overall best functional is B3LYP, which performs neither exceptionally well nor poorly in any of our previous benchmarks. It offers an overall MAE of 0.28 V and MAEs of 0.34 and 0.21 V for *E*(M^+^/M*) and *E*(M*/M^–^), respectively. B2PLYP and M06 follow closely with accuracies identical to that of B3LYP within the margin of error. The unexpectedly good performance of B3LYP also indicates that error compensation may still be present. Therefore, we explored ways to refine our prediction approach. The evident option is to improve the *E*_abs_ approximation of *E*_0,0_, as we have seen that even a simple scaling of the vertical absorption energies offers a huge leap in consistency (compare Figures S7 and S8 where *E*_0,0_ = *E*_abs_ with Figures S9 and S10 where *E*_0,0_ = 0.91 × *E*_abs_). This type of scaling does not influence the differences in TDDFT performance of the functionals, which we have already explored in a previous paper.^10^ We could use the optimized mean wavelength scaling factors for each functional to alleviate this difference. However, it would introduce additional functional dependent parameters that we wish to avoid.

**Figure 7:**
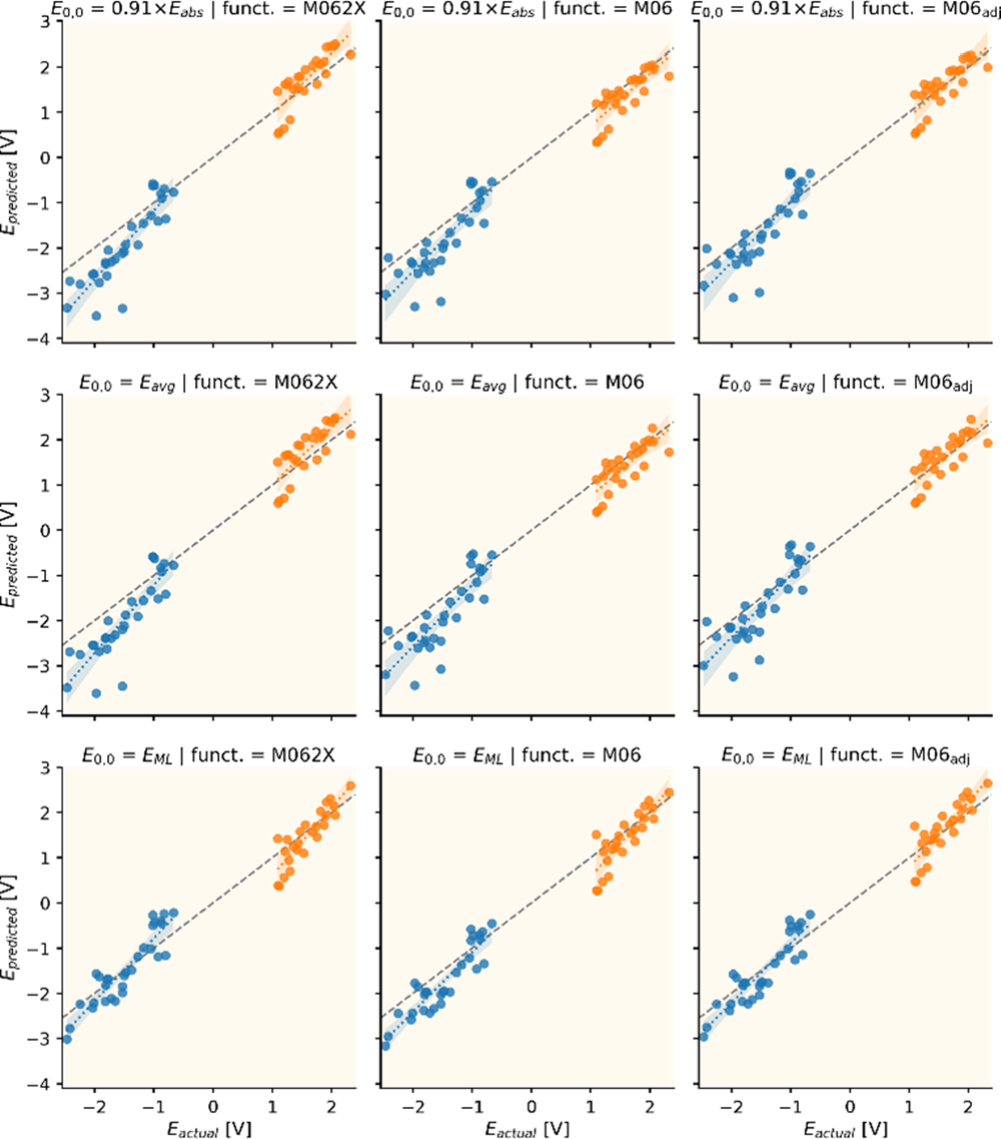
Comparison of different *E*_0,0_ approximations (rows) for the M062X and M06 functionals (columns) together with a third column where the universal ground state potential shift of 0.2 V is applied to the M06 predictions (see text). The dashed lines in gray indicate perfect match. The blue and orange colors correspond to the *E*(M^+^/M*) and *E*(M*/M^–^) potentials, respectively.

One option to obtain a better *E*_0,0_ estimate is to calculate the first excited state with vibrational resolution for all molecules. Such calculations, however, involve layers of approximations (that may break down at high Stokes shifts) to make them feasible for larger molecules.^43^ Therefore, they are not adequate for cost-effective calculations. A conceptually simpler method is to calculate the first state in the emission spectra and average it with *E*_abs_. This averaging (*E*_avg_) approach is analogous to how *E*_0,0_ is determined from measured spectra. It involves costly excited state optimizations, but we have found that nine optimization steps are sufficient for all our molecules to achieve reasonably converged emission spectra. Still, we only analyze here the M062X and M06 functionals in the following, as M062X offers accurate ground state potentials while M06 yields accurate *E*_abs_ values (i.e., it has the best TDDFT performance among the hybrid functionals considered here).^10^ We do not consider double hybrids here, as we have seen so far that their significantly larger computational cost does not translate to improved accuracy. In addition, technical reasons (lack of analytical gradients) also rule out such calculations. The results in Figure 7 however, indicate that this *E*_avg_ approach yields results almost identical to that of the scaled absorption (0.91 × *E*_abs_) estimate. Therefore, using this 0.91 universal empirical scaling parameter instead of calculating the emission spectra is recommended to save significant computational effort.

### *E*_0,0_ from ML

**Figure 8:**
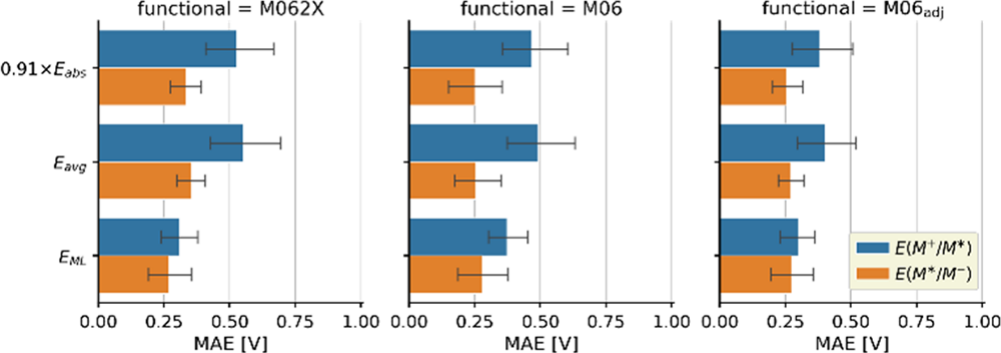
Comparison of the errors of the excited state potentials calculated using the scaled absorption, averaging, and ML approximations for *E*_0,0_.

We have also set out to explore the possibility of avoiding TDDFT calculations. To this end, we have used the freely accessible version of the Deep4Chem database to fit an ML model that predicts *E*_0,0_ values. The subset of the database relevant for our purpose contains absorption and emission values for about 1700, 2300, and 600 different organic chromophores in acetonitrile, DCM, and *N*,*N*-Dimethylformamide solvents (these are the solvents of our benchmark set of organic PC molecules; see Table S4), respectively. We have calculated *E*_0,0_ via averaging the tabulated absorption and emission maxima and fitted three deep learning models with the same architecture for the three solvents. Additional details about the model are included in the Computational Methods section. The predictions at the bottom of Figure 7 show the excited state redox potentials obtained by mixing the ML predictions for *E*_0,0_ (*E*_ML_) with the ground state potentials from the three DFT methods. The very good performance is apparent, especially for *E*(M^+^/M*), where both the accuracy and variance are noticeably better than the approaches based on exclusively DFT. Furthermore, the combination of *E*_0,0_ from ML with M062X ground state redox potentials yields the lowest MAEs (Figure 8) for not only *E*(M^+^/M*) but also *E*(M*/M^–^) without the need for any empirical adjustment. A better estimation for the ground state potential (M06_adj_ + ML) slightly improves the results; however, this requires the use of one (0.2 V) or two (Figure 4) shift parameters. For a DFT-only protocol, the use of parameters cannot be avoided to achieve even comparable results: the ground state redox potentials must be shifted (0.2 V is used for M06_adj_ in Figures 7 and 8) and a scaling of 0.91 must be applied to the *E*_abs_ values. Note that the scaling parameter can be exchanged for additional computational effort, i.e., via the calculation of *E*_avg_.

### Prediction of the Range of Redox Potentials for PC Scaffolds

Knowledge about the redox window available for each type of PCs is crucial for planning organic synthesis, so we use this protocol to further evaluate our best performing approaches. Our benchmark set contains nine different molecular scaffolds (Scheme 3); however, there are several molecules where either the *E*(M^+^/M*) or the *E*(M*/M^–^) potential is not provided in the literature. The missing data make evaluating the accuracy of our models less impactful for some scaffold/potential combinations; however, it also points out the advantage of calculations to predict missing data. For an overview of the reference data, see Table S5.

**Figure 9:**
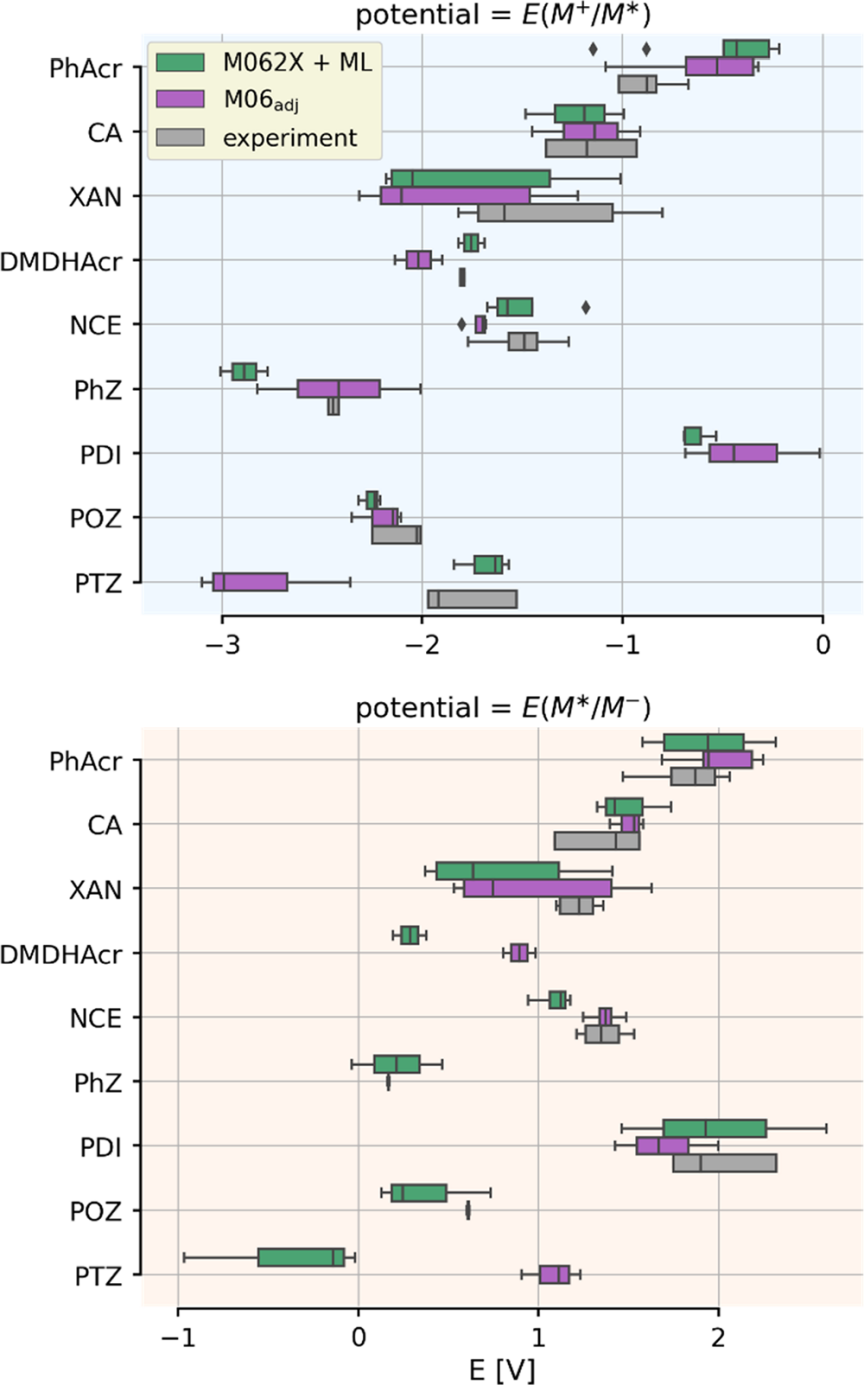
Box and whisker plots showing the predicted and measured ranges of excited state redox potentials covered by the different molecular scaffolds shown in Scheme 3. Note that there are cases where reference data are not available. PhAcr: phenylacridinium, CA: cyanoarene, XAN: xanthene, DMDHAcr: dimethyl dihydroacridine, NCE: naphthochromenone, PhZ: phenazine, PDI: perylene diimide, POZ: phenoxazine, PTZ: phenothiazine.

Figure 9 shows the redox ranges calculated using the M062X + ML and the adjusted M06 approaches together with the experimental data. With this combination, we can showcase two different strategies: one is fully based on DFT, while the other combines ground state DFT with ML. We can see that the related boxes are very close to each other for the majority of the scaffolds. In particular, we point out the very nice agreement between theory and experiment for acridinium (PhAcr) and cyanoarenes (CA) families, which are presently the most popular choice in photocatalysis.^44^ However, a few cases warrant further discussions: (1) the *E*(M*/M^–^) potential of the dimethyl dihydroacridines is missing from the reference dataset and the two computational approaches provide predictions about 0.8 V apart, (2) the mean of the *E*(M^+^/M*) potential of the phenazines is reproduced much better by M06_adj_, but the variance is considerably larger than it is in the reference, (3) the difference between the DFT and DFT + ML approaches in the prediction of both potentials for phenothiazines is also noticeable; there are no experimental data for *E*(M*/M^–^), but M062X + ML is better at reproducing the *E*(M^+^/M*) potential. Based on these results, both computational methods offer good reliability for most molecular scaffolds and can be recommended for general use in photocatalysis.

## Conclusions

In this work, we have aimed to assess computational approaches that can be used to predict excited state redox potentials for our representative set of organic PCs with reasonable accuracy. As excited state redox potentials are obtained from the corresponding ground state redox potentials and the 0–0 transition energies (*E*_0,0_), we evaluated the performance of selected DFT functionals for both terms. We also introduced ML to estimate the *E*_0,0_ component of the excited state redox potentials as an alternative approach to save computational effort and to simplify the process. The following conclusions can be drawn:

1) The tested functionals predict the ground state one-electron oxidation and reduction potentials of the set of PCs with an overall 0.38 V MAE. This MAE can be lowered to 0.22 V if the systematic underestimation is corrected with a potential shift. The best performing functional is M062X.
2) Further analysis has revealed that a considerable amount of error can be attributed to solvent effects, which can be compensated by functional-dependent shifts. Applying these shifts yields very satisfactory and quite uniform DFT performances (cf. Figures 2 and 4). Similar performances are obtained by employing a uniform, optimized shift value of 0.2 V.
3) To estimate *E*_0,0_, various options have been considered.
  a. Conceptually, the simplest approach is when the first vertical excitation energy obtained by TDDFT (*E*_abs_) approximates *E*_0,0_. This approach combined with the original ground state DFT redox potentials yields inconsistent results. For example, the two types of potentials are predicted with significantly different accuracies. This is due to the mixing of errors from the ground and excited state components of the excited state redox potentials.
  b. The results are improved when the *E*_abs_ values are corrected by the empirical 0.91 scaling factor to approach *E*_0,0_.
  c. A conceptually more appropriate but computationally more demanding procedure has also been assessed, when *E*_0,0_ is approximated as the average of the first vertical absorption and emission energies. This approach has not proved to be more accurate than using the scaled *E*_abs_ approximation.
  d. An important conclusion is that any approach based on exclusively DFT needs to be adjusted by at least one added empirical parameter.
4) A significant improvement can be achieved in terms of both accuracy and computational effort if we employ ML to predict the *E*_0,0_ values. We have shown the potential of ML in two different approaches: one where we do not use empirical parameters (M062X + ML) and the other where only the solvation related 0.2 V shift is employed (M06_adj_ + ML). These two approaches yield similar MAE around 0.3 V, which is very close to the accuracy of the ground state redox potential prediction.
5) As a demonstration, we have also analyzed the performance of adjusted DFT and DFT + ML protocols via the calculation of excited state redox ranges for the different organic PC scaffolds, which is an important task in PC research. Both methods provide reliable predictions, which shows the power of approaches based on exploiting the strengths of DFT and compensating for its shortcomings via simple empirical corrections or ML.
6) Regarding the ML model, we have employed a simple neural network setup. This implies that there is considerable room for improvements in our strategy. In addition, approaches when all components are predicted by ML seem also very promising in the light of our present results. Improving the ML part of this DFT + ML technique and to undertake fully ML approaches for this and similar tasks is already underway in our group.

## Supporting Information Available

Plot showing the correlation between *E*_abs_ and *E*_0,0_; analysis of solvent effects; predicted ground state redox potentials employing different corrections; predicted excited state redox potentials employing various corrections; list of solvents; reference data used for the ground and excited state potentials; and analysis of the ML model (PDF)
