# Supplementary material for: Prediction of Redox Power for Photocatalysts: Synergistic Combination of DFT and Machine Learning
Source: J Chem Theory Comput. 2023 Jun 29;19(13):4125–35. doi: 10.1021/acs.jctc.3c00286 (PMC10339717; doi:10.1021/acs.jctc.3c00286)
Supplement: Supplementary file 1 — ct3c00286_si_001.pdf [file ct3c00286_si_001.pdf]

Prediction of Redox Power for Photocatalysts: Synergistic Combination of DFT and Machine Learning

Péter P. Fehér*[1], Ádám Madarász[1], and András Stirling*[1,2]

[1]Institute of Organic Chemistry, Research Centre for Natural Sciences, Magyar tudósok körútja. 2, Budapest, 1117, Hungary

[2]Department of Chemistry, Eszterházy Károly University, Leányka u. 6, 3300 Eger, Hungary

E-mail: feher.peter@ttk.hu, stirling.andras@ttk.hu

List of contents

Figs. S1-2	Relation between $E_{0,0}$ and E_{abs}	Page S3
Figs. S3	The distribution of absolute reaction solvation energies	Page S4
Tables S1-2	Redox potentials for the eosins using different numbers of counterions	Page S4
Figs. S4-5	Ground state redox potentials when both shift and scaling corrections are applied to the predictions	Page S5
Fig. S6	MAEs of the different shifts applied to the predicted ground state potentials	Page S6
Table S3	The MAEs for the shifts in Fig. S6	Page S6
Figs. S7-8	Excited state redox potentials without the use of any adjustment	Page S7
Figs. S9-10	Excited state redox potentials using the $E_{0,0} = 0.91 \times E_{abs}$ approximation	Page S8
Figs. S11-12	Excited state redox potentials using the $E_{0,0} = 0.91 \times E_{abs}$ approximation and the universal ground state potential shift of 0.2 V	Page S9
Table S4	Solvents used for the molecule types	Page S10
Table S5	Reference data used for the ground and excited state potentials	Page S11
Figs. S13-17	Analysis of the machine learning model	Page S12

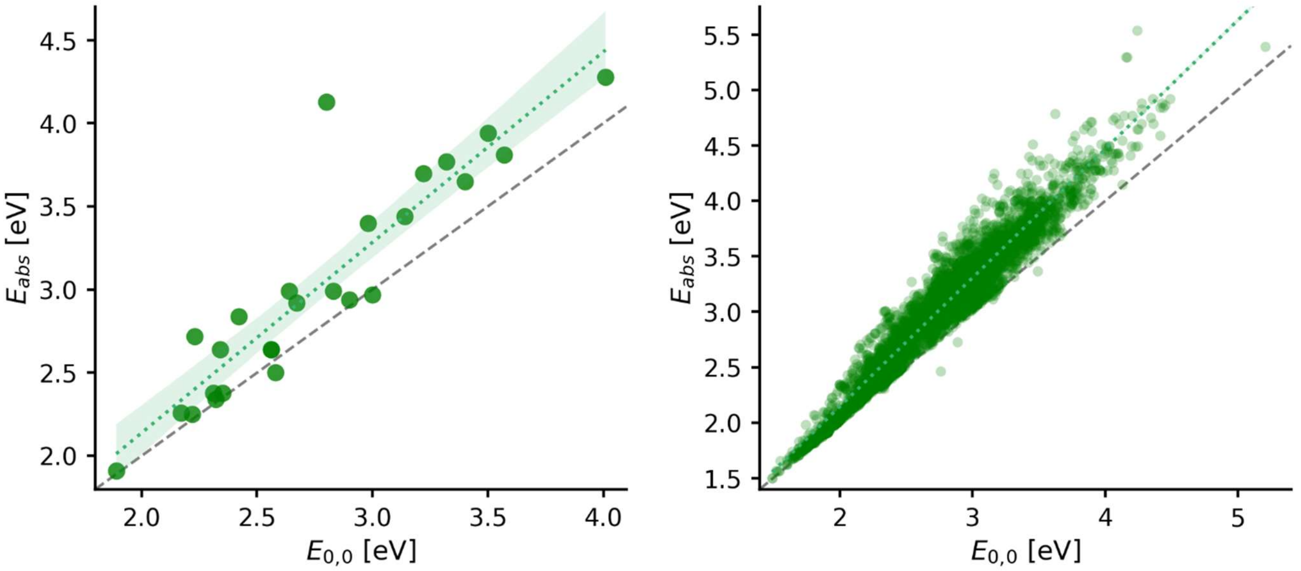

Figure S1. The relation between the 0-0 energy and the lowest energy maximum in the absorption spectra. The data on the left and right are taken from the review paper of Nicewicz et al.[1] and the Deep4Chem database,[2] respectively. The MAE of approximating $E_{0,0}$ with E_{abs} is 0.26 eV (left, 0.22 eV without the outlier) and 0.27 eV (right).

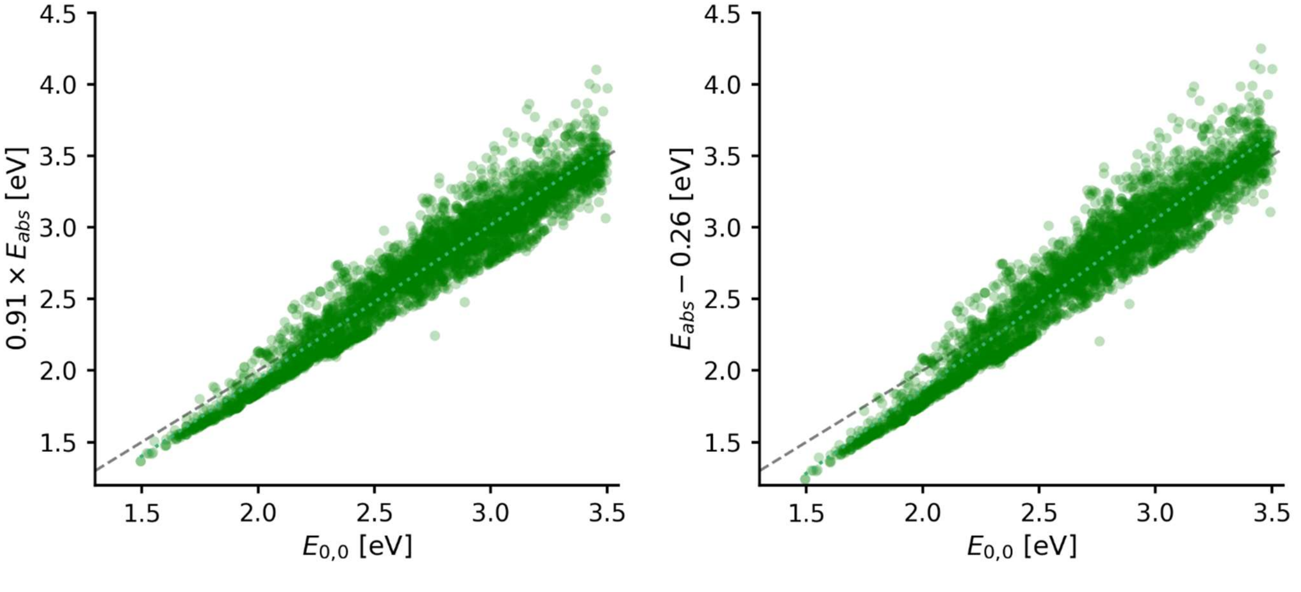

Figure S2. The approximation of the 0-0 energy with scaled (left) and shifted (right) lowest energy absorption maxima (E_{abs}). The 0.91 scaling and −0.26 eV shift yield MAEs of 0.11 and 0.14 eV, respectively. Similar, slightly larger mean absolute deviation of 0.15 V has been reported by Blase et al using sophisticated hybrid schemes to directly calculate $E_{0,0}$.[3] Note that the $E_{0,0}$ values above 3.5 eV (below 350 nm) were not considered here as they are well outside the visible range.[4]

[1] N. A. Romero and D. A. Nicewicz, *Chem. Rev.*, **2016**, *116*, 10075–10166
[2] J. F. Joung, M. Han, M. Jeong and S. Park, *Sci. Data*, **2020**, *7*, 295.
[3] Jacquemin, D.; Duchemin, I.; Blase, *J. Chem. Theory Comput.*, **2015**, *11*, 5340–5359.
[4] For details, see https://github.com/PeterF1234/DFT-ML-excited-state-redox

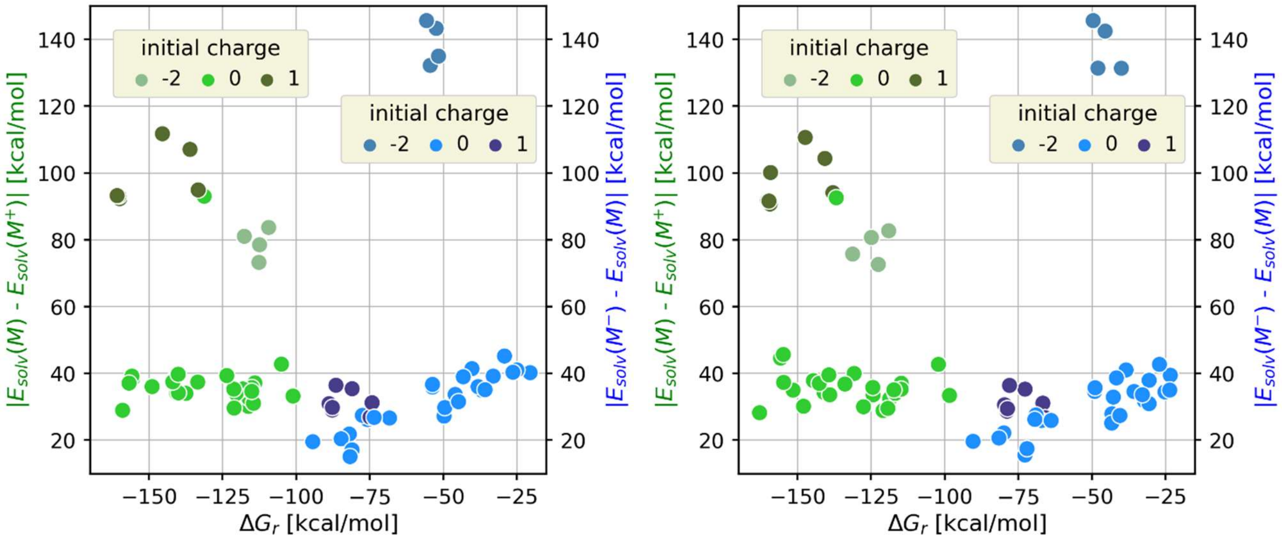

Figure S3. The distribution of absolute reaction solvation energies ($|\Delta E_{solv}|$) with respect to the reaction free energies (ΔG_r) calculated with the ωB2PLYP (left) and DSD-BLYP (right) functionals.

Table S1. M062X calculated $E(M/M^-)$ potentials for the eosins using different numbers of counterions.

	eos²⁻	*Na(eos)⁻*	*Na₂(eos)*	*expt.*
EOS_1	−2.10	−1.85	−1.62	−1.11
EOS_2	−1.99	−1.75	−1.48	−0.99
EOS_3	−2.10	−1.83	−1.60	−1.08
EOS_Y	−1.92	−1.72	−1.45	−1.00

Table S2. M062X calculated $E(M^+/M)$ potentials for the eosins using different numbers of counterions.

	eos²⁻	*Na(eos)⁻*	*Na₂(eos)*	*expt.*
EOS_1	0.13	0.20	0.46	0.39
EOS_2	0.21	0.30	0.54	0.54
EOS_3	0.17	0.25	0.55	0.48
EOS_Y	0.45	0.52	0.90	0.77

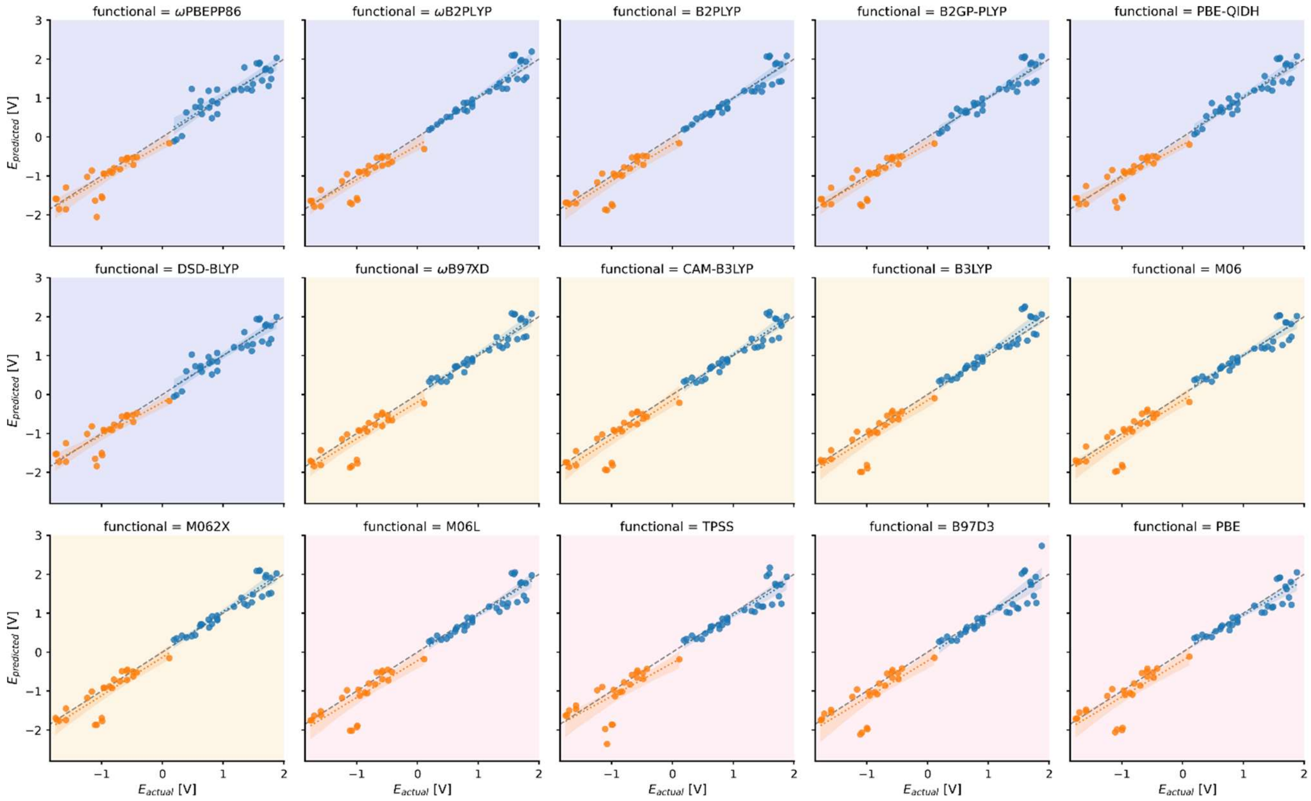

Figure S4. Calculated vs reference ground state redox potentials when both shift and scaling corrections are applied to the predictions.

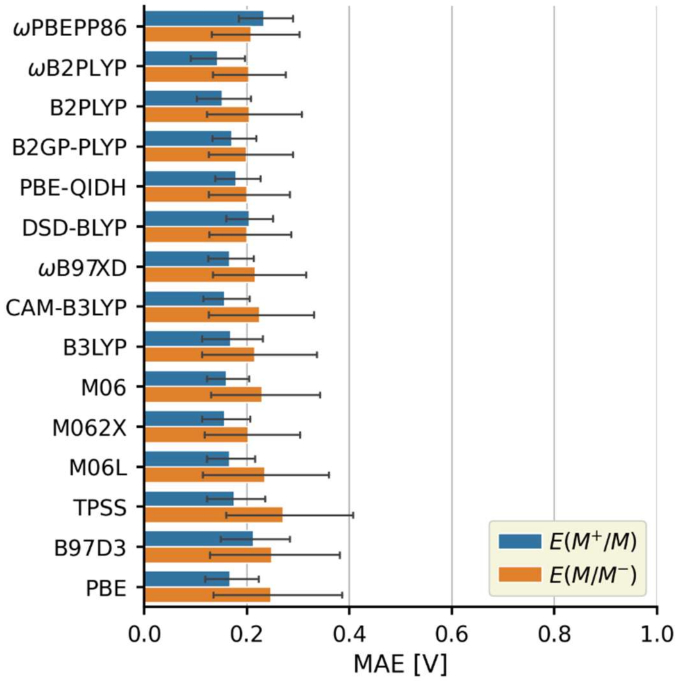

Figure S5. The MAEs of ground state redox potential prediction when both shift and scaling corrections are employed.

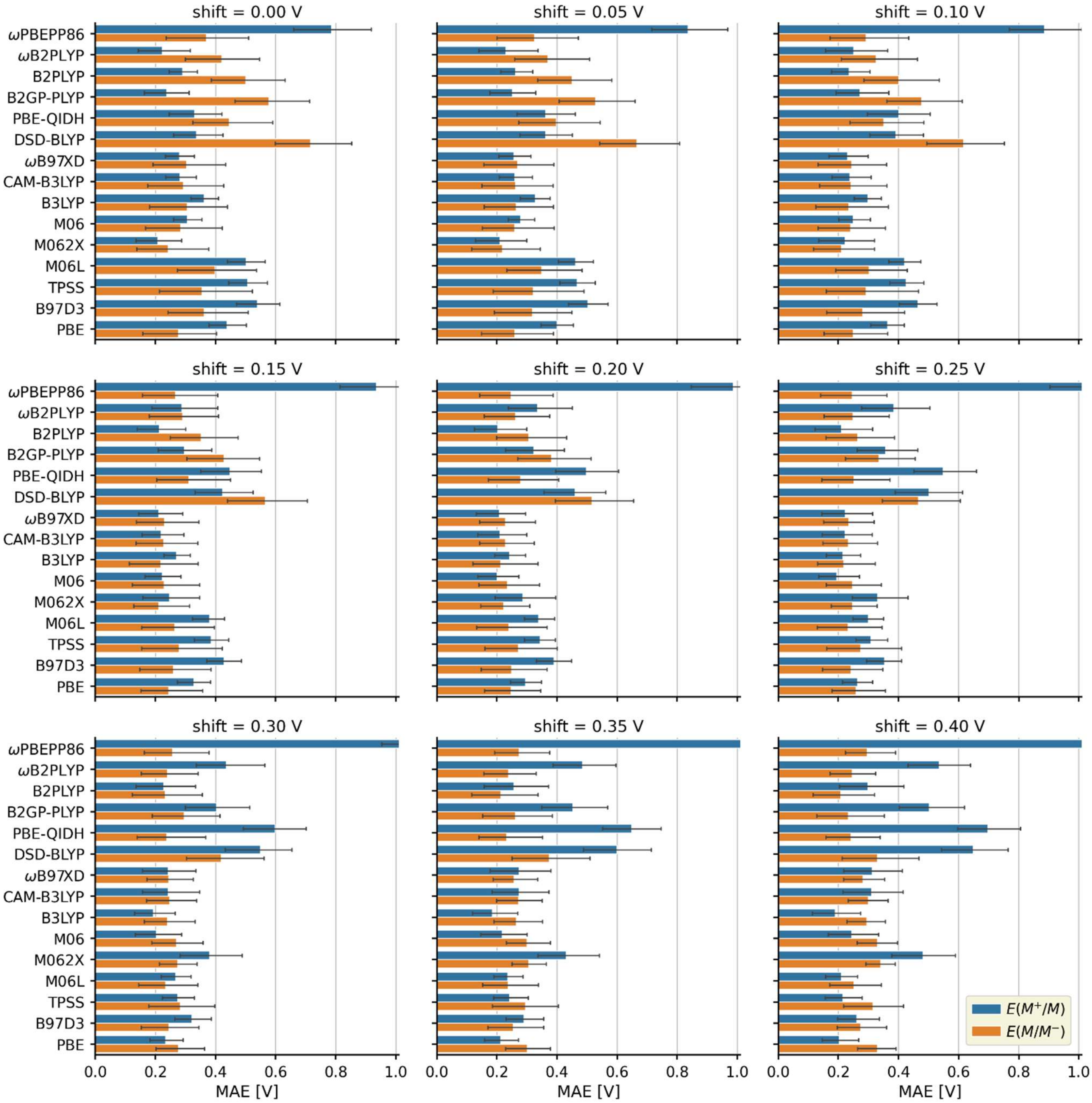

Figure S6. The MAEs of each functional when different shifts are applied to the predicted ground state potentials.

Table S3. MAEs calculated for all and hybrid functionals at different shift values in Fig S6.

shift	*MAE*	*MAE*
0.00 V	0.382 (±0.010)	0.287 (±0.015)
0.05 V	0.358 (±0.010)	0.261 (±0.014)
0.10 V	0.339 (±0.010)	0.242 (±0.014)
0.15 V	0.326 (±0.010)	0.229 (±0.014)
0.20 V	0.320 (±0.011)	0.227 (±0.014)
0.25 V	0.321 (±0.011)	0.237 (±0.014)
0.30 V	0.328 (±0.011)	0.253 (±0.014)
0.35 V	0.341 (±0.011)	0.278 (±0.014)
0.40 V	0.360 (±0.011)	0.308 (±0.014)

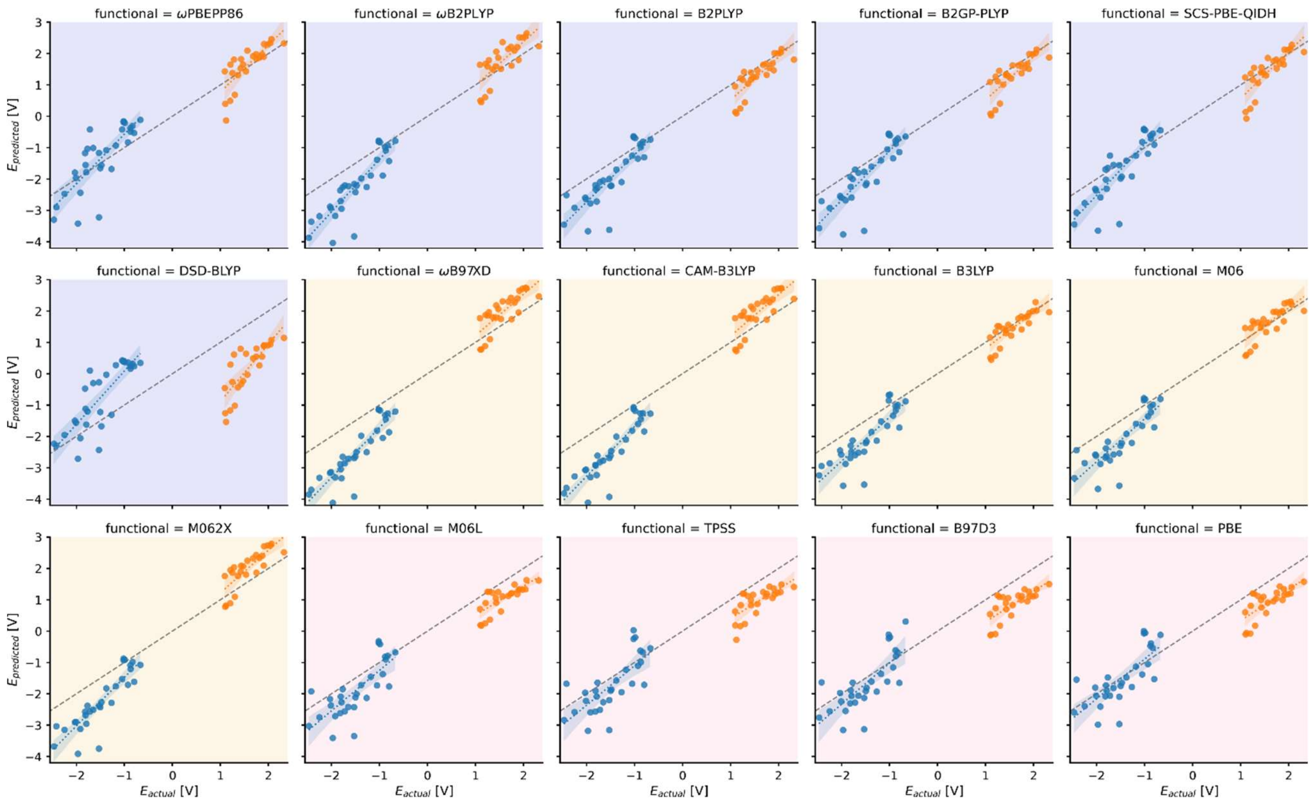

Figure S7. Calculated vs reference excited state redox potentials without any adjustment.

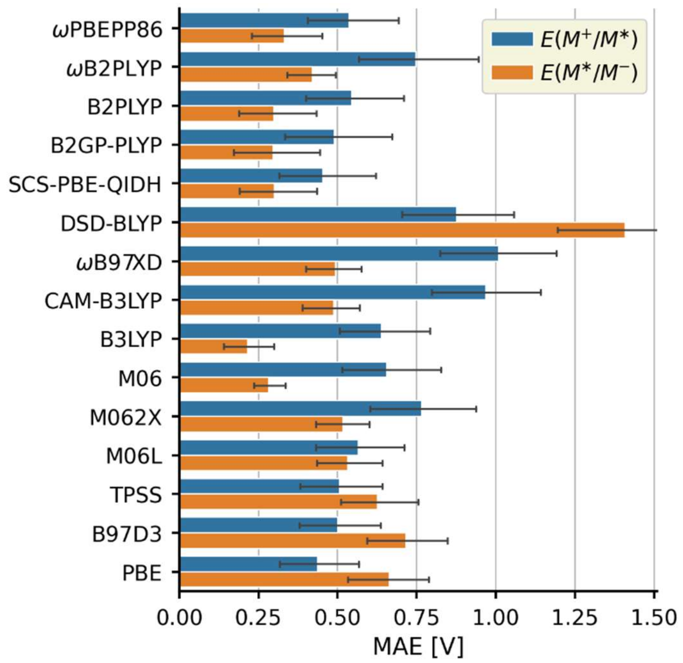

Figure S8. The MAEs of excited state redox potential prediction without any adjustment.

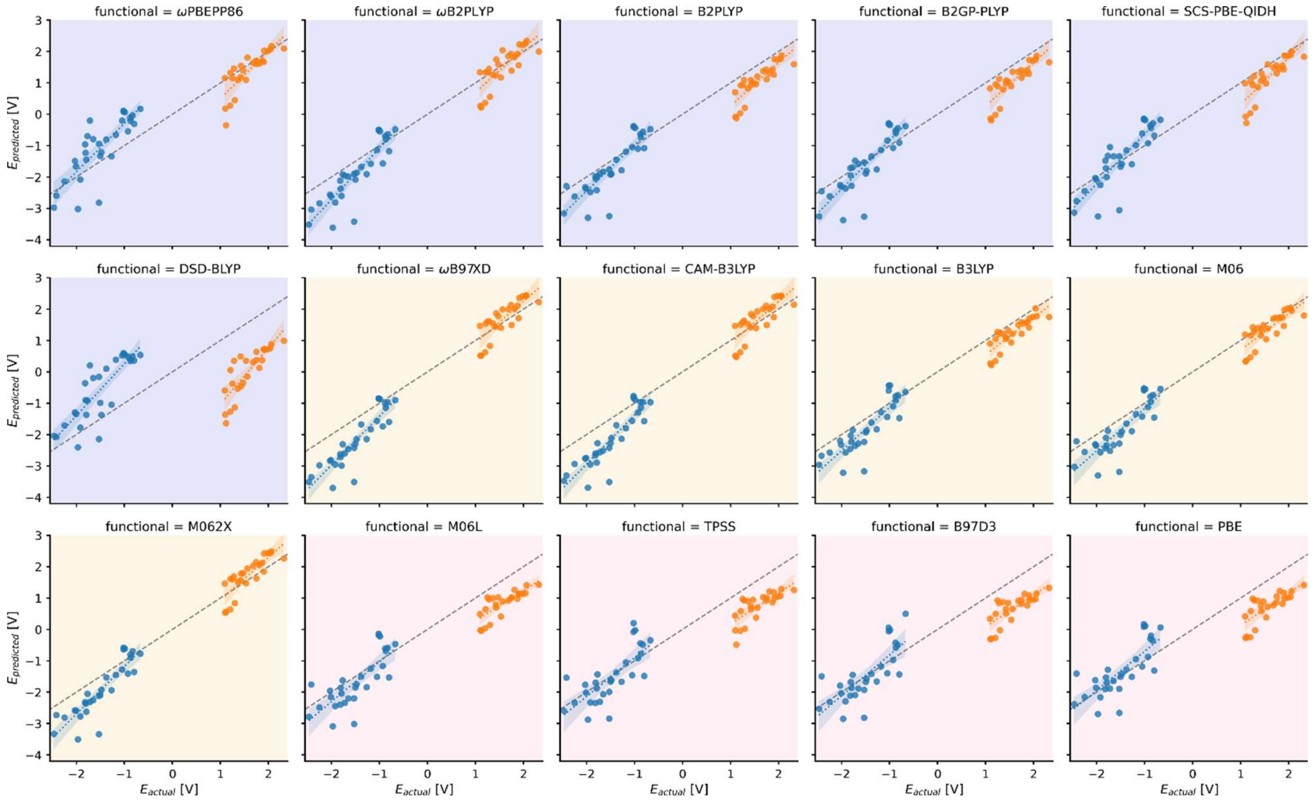

Figure S9. Calculated vs reference excited state redox potentials using only the $E_{0,0}$ = 0.91×E_{abs} approximation.

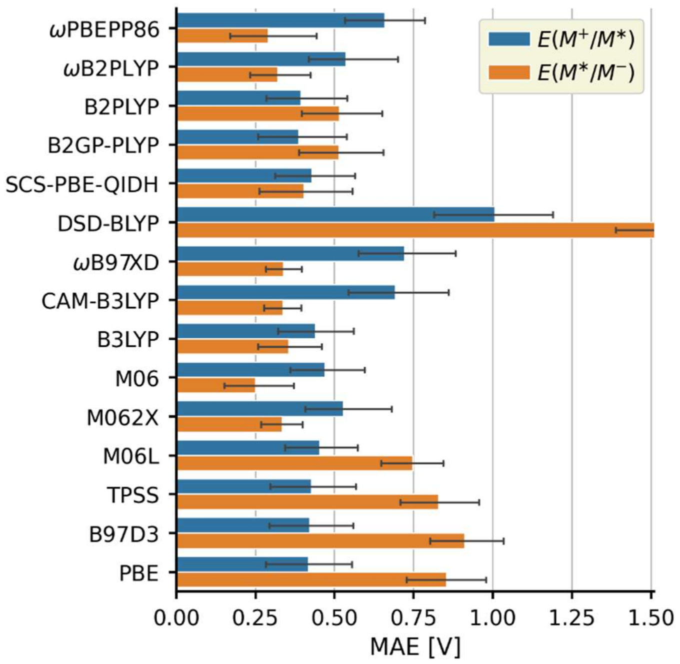

Figure S10. The MAEs of excited state redox potential prediction using the $E_{0,0}$ = 0.91×E_{abs} approximation.

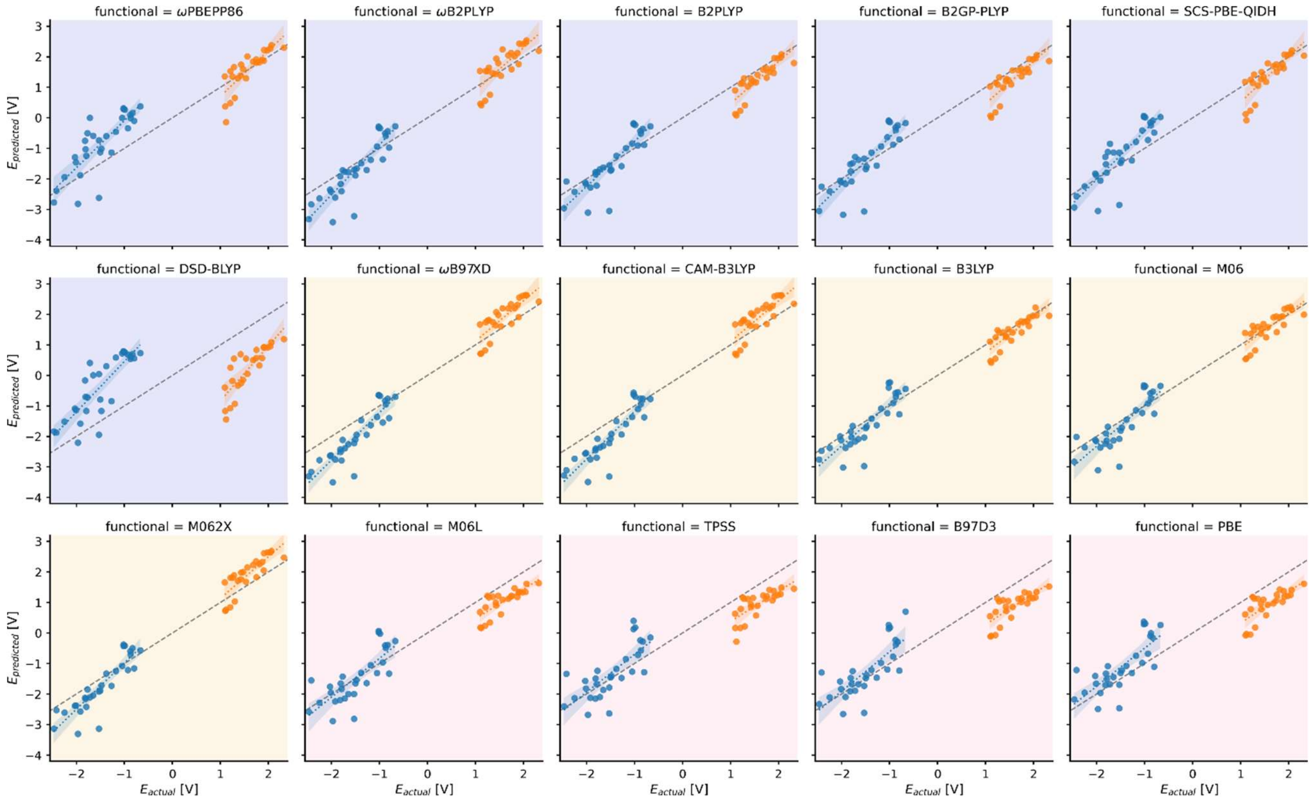

Figure S11. Calculated vs reference excited state redox potentials using the $E_{0,0} = 0.91 \times E_{abs}$ approximation and the universal ground state potential shift of 0.2 V.

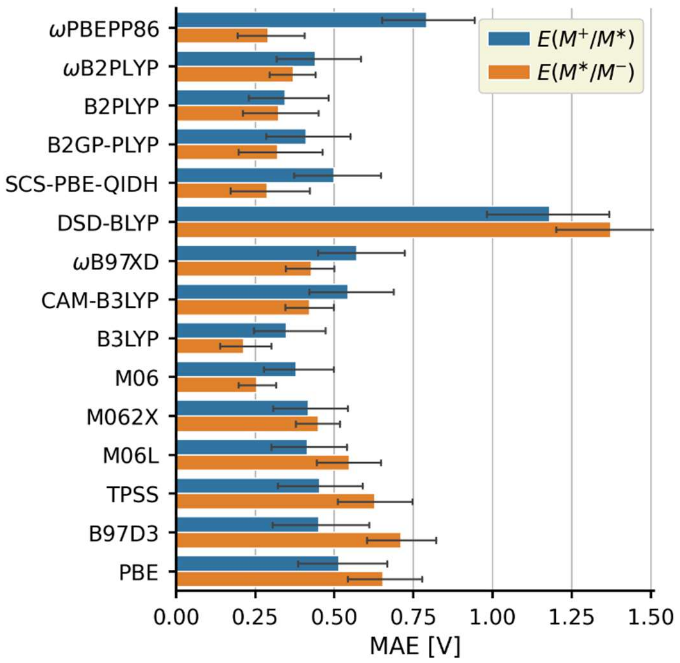

Figure S12. The MAEs of excited state redox potential prediction using the $E_{0,0} = 0.91 \times E_{abs}$ approximation and the universal ground state potential shift of 0.2 V.

Table S4. Solvents used for the molecule types. The entries with parentheses indicate cases where different solvents were used for ground state and excited state (TDDFT, in parentheses) calculations. In the ML models acetonitrile and *N*,*N*-Dimethylformamide were used instead of benzonitrile and *N*,*N*-Dimethylacetamide.

molecule group	solvent
BF_3_Acr	dichloromethane
BOH_Acr	dichloromethane
Mes-Acr	benzonitrile (acetonitrile)
Ph_Acr	acetonitrile
Me_2_Acr	*N*,*N*-Dimethylformamide
CA	acetonitrile
Eos	acetonitrile
Rh_B	acetonitrile (ethanol)
Rh_6G	acetonitrile (methanol)
NCE	acetonitrile
PA	*N*,*N*-Dimethylformamide
PDI	dichloromethane
POZ	*N*,*N*-Dimethylacetamide
PTZ	*N*,*N*-Dimethylacetamide

Table S5. Reference data used for the ground and excited state potentials of the OPC molecules in our dataset. All values are relative to the saturated calomel reference electrode (SCE).

molecule	$E(M^+/M)$ [V]	$E(M/M^-)$ [V]	$E(M^+/M^*)$ [V]	$E(M^*/M^-)$ [V]	source
BF3-Acr_m	1.69	-0.7	-0.88	1.87	10.1021/acsomega.8b03290
BF3-Acr_o	1.78	-0.97	-0.87	1.68	10.1021/acsomega.8b03290
BF3-Acr_p	1.71	-0.8	-0.83	1.74	10.1021/acsomega.8b03290
BOH-Acr_m	1.59	-0.67	-0.99	1.91	10.1021/acsomega.8b03290
BOH-Acr_o	1.6	-0.58	-1.02	2.04	10.1021/acsomega.8b03290
BOH-Acr_p	1.55	-0.59	-1.02	1.98	10.1021/acsomega.8b03290
CA_1	1.49	-1.24	-1.18	1.43	10.1021/jacs.8b08933
CA_2	1.3	-1.59	-1.38	1.09	10.1021/jacs.8b08933
CA_3	1.79	-1.16	-0.93	1.56	10.1021/jacs.8b08933
Eos_1	0.39	-1.11	-1.82	1.1	10.1002/cptc.202000153
Eos_2	0.54	-0.99	-1.65	1.2	10.1002/cptc.202000153
Eos_3	0.48	-1.08	-1.72	1.12	10.1002/cptc.202000153
Eos_Y	0.77	-1	-1.53	1.3	10.1002/cptc.202000153
Me2-Acr_2	0.76		-1.79		10.1002/anie.201910828
Me2-Acr_3	0.9		-1.81		10.1002/anie.201910828
Mes-Acr_1	1.88	-0.49	-0.67	2.06	10.1021/ja038656q
NCE_1	1.75	-1.74	-1.27	1.28	10.1002/anie.201912455
NCE_2	1.47	-1.76	-1.5	1.21	10.1002/anie.201912455
NCE_3	1.64	-1.7	-1.48	1.42	10.1002/anie.201912455
NCE_4	1.35	-1.59	-1.77	1.53	10.1002/anie.201912455
PA_1	0.19		-2.47		10.1126/science.aaf3935
PA_2	0.23		-1.64		10.1126/science.aaf3935
PA_3	0.32		-2.42		10.1126/science.aaf3935
PDI_1		-0.58255		1.75	10.1039/c9cc07040k
PDI_2		-0.42067		1.9	10.1039/c9cc07040k
PDI_3		0.112162		2.32	10.1039/c9cc07040k
POZ_1	0.63		-2.03		10.1021/jacs.7b12074
POZ_3	0.65		-2.25		10.1021/jacs.7b12074
POZ_5	0.62		-2.01		10.1021/jacs.7b12074
PTZ_1	0.815		-1.97		10.1021/jacs.5b13455
PTZ_2	0.903		-1.53		10.1021/jacs.5b13455
PTZ_3	0.902		-1.92		10.1021/jacs.5b13455
Ph-Acr_1		-0.83		1.47	10.1021/acscatal.9b03606
Ph-Acr_2		-0.48		1.81	10.1021/acscatal.9b03606
Rh_6G	1.4	-0.95	-0.8	1.25	10.1002/cctc.201800971
Rh_B	1.18	-0.87	-1.05	1.36	10.1002/ejic.201600755

Analysis of the machine learning model

In the following, we present the results of model training for the three solvents. Fig. S14 shows the accuracy of predictions for the test sets (we used a 80:20 train-test split) and the OPC molecules in their corresponding solvents. Additional performance metrics are provided (or can be generated) in the Jupyter notebook on GitHub. We have also assessed the similarities between the molecules in the Deep4Chem database and those in our benchmark set. To this end, we have calculated Tanimoto coefficients (using the default RDKit fingerprint) and performed maximum common substructure (MCS) analysis (with complete ring matching turned on) for every pair of OPC and database molecules in the three solvents using RDKit. The distributions of the Tanimoto coefficients are shown in Figs. S15-17, while the MCS results (MCS lengths and matched substructures) can be accessed in the Jupyter notebook on GitHub. The results indicate that the similarities between the OPC set and the training data are rather low, except for xanthenes (the two rhodamines are in the dataset). A closer inspection of the most similar structures and the MCS analysis (e.g., Fig. S18), however, indicates that the main chromophore backbones are included in the training set. The relatively low similarities are therefore not a major issue in this case. Nevertheless, there is certainly room for improvement here which further emphasizes the potential of using ML to predict properties that are difficult to obtain via ab initio calculations. Fine tuning the ML model is outside of the scope of the present work and will be investigated in a following study.

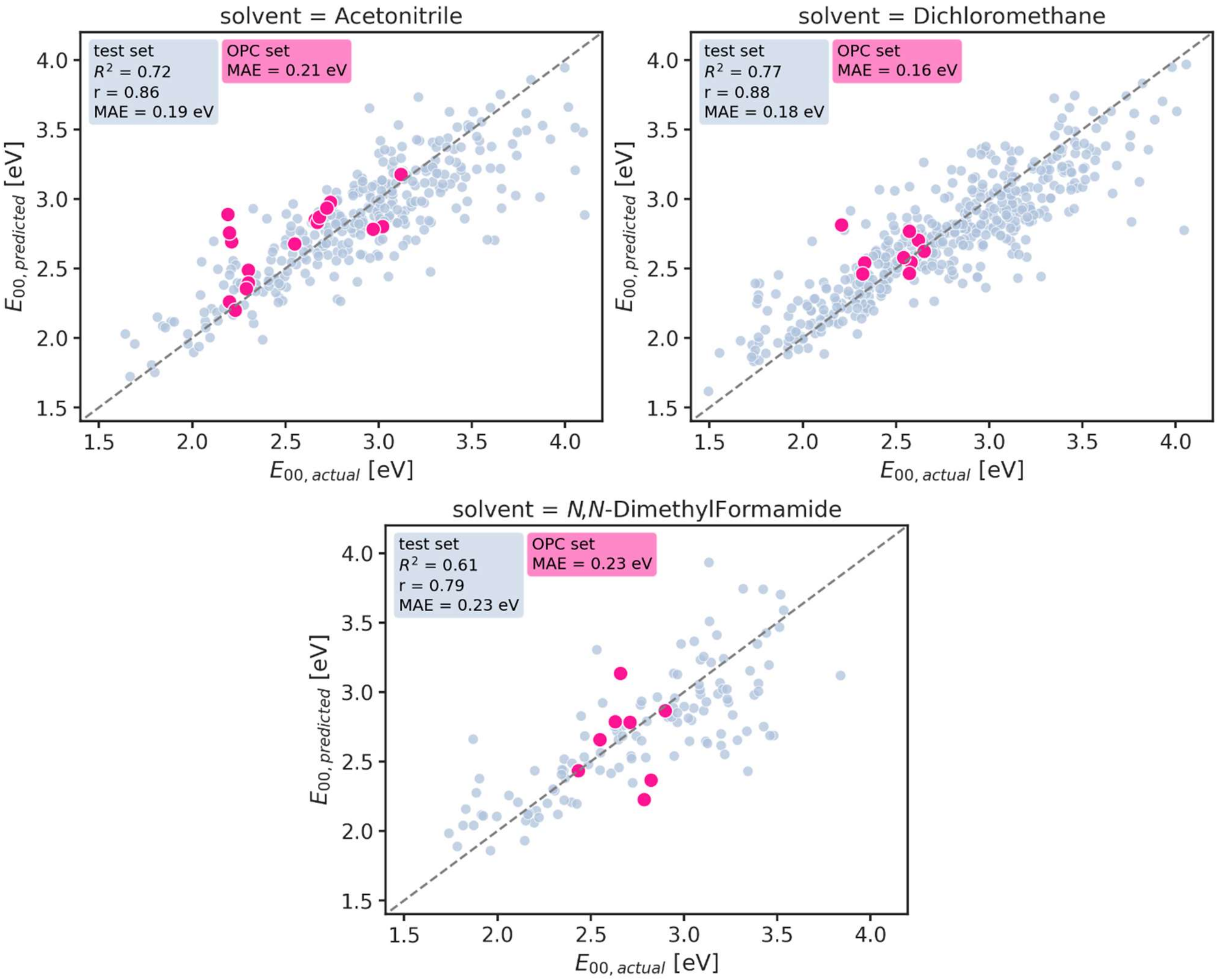

Figure S13. Predicted vs actual $E_{0,0}$ values for the test set (blue points) and the OPC benchmark set (magenta points) in the three solvents. Note that the coefficient of determination and Pearson correlation is not shown for the OPC set as it is not sampled from the same population as the training set.

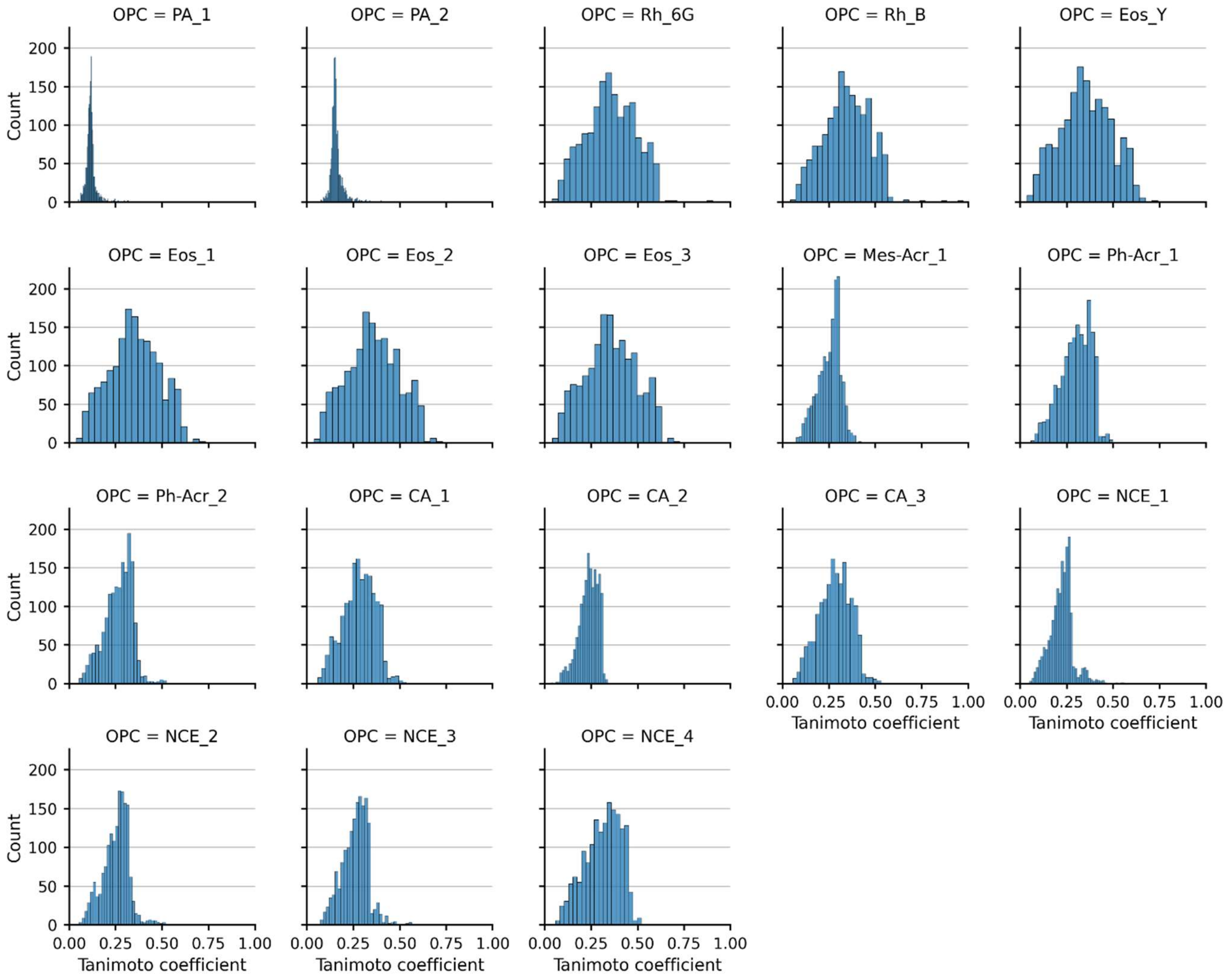

Figure S14. The distributions of molecular similarity between the individual OPCs and the molecules in the Deep4Chem database for the acetonitrile solvent.

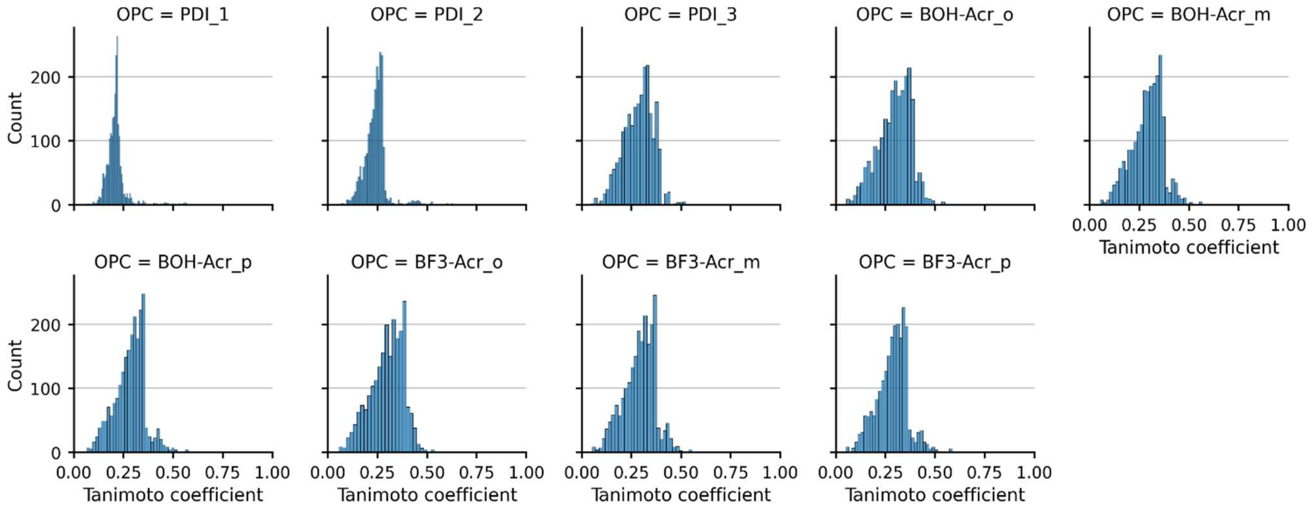

Figure S15. The distributions of molecular similarity between the individual OPCs and the molecules in the Deep4Chem database for the dichloromethane solvent.

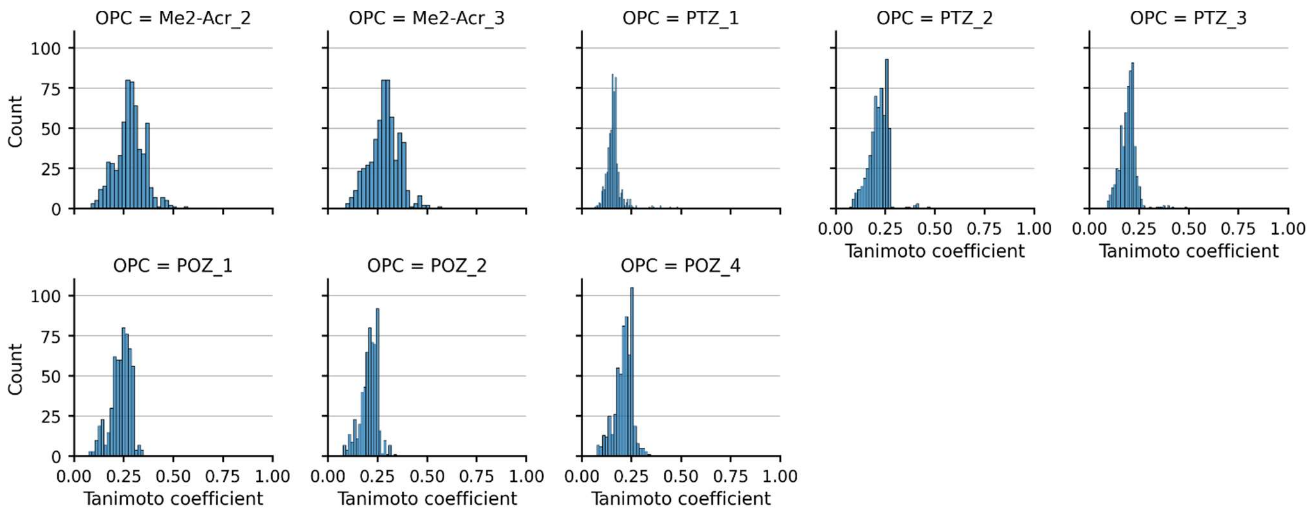

Figure S16. The distributions of molecular similarity between the individual OPCs and the molecules in the Deep4Chem database for the DMF solvent.

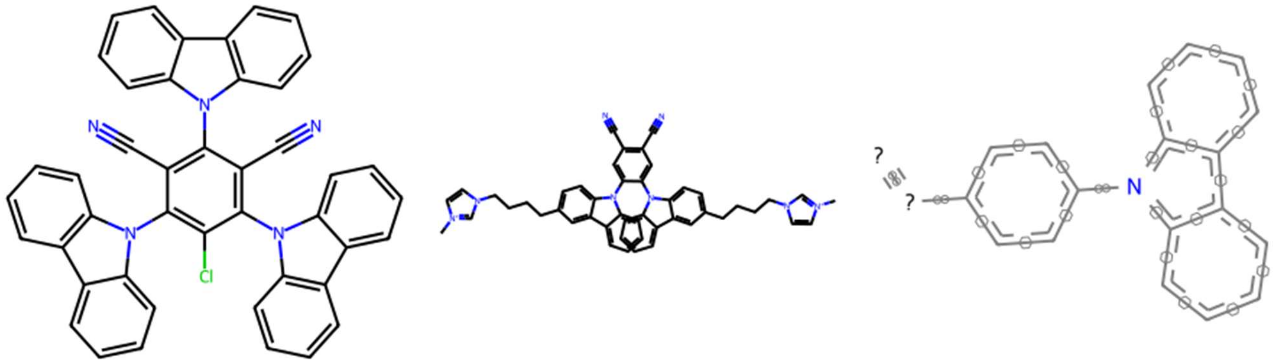

Figure S17. The CA_3 molecule (left), together with the most similar molecule (T = 0.53) in the Deep4Chem database and their MCS. Additional examples can be generated using the Jupyter notebook at GitHub.
